# Context Drives Diversification of Monocytes and Neutrophils in Orchestrating the Tumor Microenvironment

**DOI:** 10.3389/fimmu.2019.01817

**Published:** 2019-08-16

**Authors:** Juhee Jeong, Yoorock Suh, Keehoon Jung

**Affiliations:** ^1^Lab of Cancer Immunology and In Vivo Imaging, Department of Biomedical Sciences, BK21 Plus Biomedical Science Project, Seoul National University College of Medicine, Seoul, South Korea; ^2^Department of Anatomy and Cell Biology, Seoul National University College of Medicine, Seoul, South Korea; ^3^Institute of Allergy and Clinical Immunology, Seoul National University Medical Research Center, Seoul, South Korea

**Keywords:** monocytes, neutrophils, tumor microenvironment, myeloid cell heterogeneity, innate immunity, cancer immunology

## Abstract

Recent preclinical/clinical studies have underscored the significant impact of tumor microenvironment (TME) on tumor progression in diverse scenarios. Highly heterogeneous and complex, the tumor microenvironment is composed of malignant cancer cells and non-malignant cells including endothelial cells, fibroblasts, and diverse immune cells. Since immune compartments play pivotal roles in regulating tumor progression via various mechanisms, understanding of their multifaceted functions is crucial to developing effective cancer therapies. While roles of lymphoid cells in tumors have been systematically studied for a long time, the complex functions of myeloid cells have been relatively underexplored. However, constant findings on tumor-associated myeloid cells are drawing attention, highlighting the primary effects of innate immune cells such as monocytes and neutrophils in disease progression. This review focuses on hitherto identified contextual developments and functions of monocytes and neutrophils with a special interest in solid tumors. Moreover, ongoing clinical applications are discussed at the end of the review.

## Monocytes: From Development to Deployment

### Monocyte Development

Monocytes originally stem from the bone marrow and constitute 10% of leukocytes in human blood and 4% of leukocytes in mouse blood, respectively (1). The development of blood monocytes is dependent on colony-stimulating factor 1 receptor, CSF-1R (also known as M-CSFR; macrophage colony-stimulating factor receptor) (1, 2). CSF-1R is a hematopoietic growth factor receptor expressed on monocytes, macrophages, dendritic cells and their progenitors (1, 2). CSF-1R interacts with its ligands CSF-1 (M-CSF) and IL-34 to regulate the development of monocytes in the bone marrow (1, 2). In mice deficient in CSF-1R and CSF-1, monocyte development is inhibited, and therefore the number of monocytes in blood is remarkably reduced (1, 2).

With knowledge of CSF-1R, it is possible to navigate the development process of monocytes. From the bone marrow, hematopoietic stem cells (HSCs) give rise to heterogeneous multipotent progenitors (MPPs) generating common myeloid progenitors (CMPs) or common lymphoid progenitors (CLPs) in a CSF-1 dependent manner (3). While lymphoid cells such as T lymphocytes, B lymphocytes, and natural killer cells are derived from CLPs, CMPs generate megakaryocyte and erythrocyte progenitors (MEPs) or granulocyte and macrophage progenitors (GMPs). Generated GMPs further go through a series of differentiation, firstly into macrophage, and DC progenitors (MDPs), then into common monocyte progenitors (cMoPs), and finally into monocytes (3). Differentiated monocytes can be divided into two main subpopulations defined as Ly6C^hi^CX_3_CR1^low^ and Ly6C^low^CX_3_CR1^hi^ cells in mice and as CD14^hi^CD16^+/−^ and CD14^low^CD16^hi^ cells in humans (4–8).

Ly6C^hi^CX_3_CR1^low^ populations (hereinafter referred to as Ly6C^hi^ monocytes) are named “classical” or “inflammatory” monocytes, whereas Ly6C^low^CX_3_CR1^hi^ populations (hereinafter referred to as Ly6C^lo^ monocytes) are named “non-classical” or “patrolling” monocytes for their preferential patrolling behavior while circulating the blood stream (9, 10). Development of Ly6C^hi^ monocytes occurs during the cMoP stage, dependent on GM-CSF, c-FLIP, IRF8, and KLF4 (10). The widely accepted hypothesis on Ly6C^lo^ monocyte differentiation is that after generation of Ly6C^hi^ monocytes from the bone marrow, a proportion of them differentiate into Ly6C^lo^ monocytes as downregulation of Ly6C and upregulation of Nr4a1, C/EBPβ, CSF-1R, and CX_3_CR1 (4, 10, 11). However, this was questioned for some time in that deletion of transcription factors KLF4 and IRF8 hinders the development of Ly6C^hi^ monocytes but not Ly6C^lo^ monocytes (12–14). This finding led to controversy on whether Ly6C^lo^ monocytes originate from Ly6C^hi^ monocytes or not. The latter argues that Ly6C^lo^ monocytes might have a distinct differentiation lineage in a Ly6C^hi^ monocyte-independent way, namely direct differentiation from cMoPs.

Single-cell RNA sequencing provided an additional clue, reasserting that Ly6C^hi^ monocyte population is the source of Ly6C^lo^ monocytes (15). Application of such advanced technology revealed that steady-state Ly6C^hi^ and Ly6C^lo^ monocytes are homogenous populations, and C/EBPβ regulates the differentiation of Ly6C^hi^ monocytes into Ly6C^lo^ monocytes (15). This is also in line with remarkable expression/function of Nr4a1 on Ly6C^lo^ monocyte development (16), as it was found that regulation of Nr4a1 is mediated by the expression of C/EBPβ and also KLF2 assisting conversion of Ly6C^hi^ monocytes to Ly6C^lo^ monocytes (12, 15–17). Besides Nr4a1, Ten-Eleven-Translation-3 (TET3), a target of hsa-miR-150, regulates differentiation of classical monocytes into non-classical monocytes in K562 human chronic myeloid leukemia and U937 human lymphoma (18). Upregulation of TET3 expression in classical monocytes following downregulation of hsa-miR150 rarely generates non-classical monocytes, but does not affect the survival of non-classical monocytes (18). Recently, single-cell RNA-seq has also led to the identification of two additional monocyte populations and their distinct relationships with other immune cells in human blood, highlighting the heterogeneity of myeloid cells (19). High-dimensional mass cytometry has further revealed heterogeneity within human non-classical monocytes, and has allowed distinguishing between two different non-classical monocyte subsets, Slan^+^ and Slan^−^, with functional differences based on Slan expression (20).

Some developed monocytes can enter non-lymphoid organs such as skin and lung without differentiation and orchestrate the physiological condition, while some portion of developed monocytes undergoes differentiation into macrophages or dendritic cells (21–24). Of note, differentiated macrophages are conventionally classified into pro-inflammatory M1 type and anti-inflammatory (pro-tumoral) M2 type, and these macrophages differentially regulate tumor progressions and metastases (25). However, this binary classification of macrophages is insufficient to represent their multifaceted and plastic functions (25). On the other hand, monocyte-derived dendritic cells have been mainly regarded as immune activators in the tumor microenvironment, recruiting and stimulating immune effector cells (26). Nevertheless, dendritic cells are also highly heterogeneous, and cancer cells can recruit the immunosuppressive subset of dendritic cells and/or suppress their anti-tumoral functions (26). All this flexibility appears in a context-dependent manner. Likewise, differing individual functions of monocytes might result from different contexts of development. While it is well-accepted that the bone marrow is the primary source of production and supply of monocytes in physiological condition (1), there is substantial controversy whether the bone marrow serves the same role in cancer-derived pathological conditions. Splenic progenitor cells are reinforced to generate monocytes during KP lung carcinoma progression, which suggests that the spleen could be a critical organ to produce and amplify monocytes (27). The pivotal role of spleen as a source of monocytes has also been highlighted in a different inflammatory condition (28). Angiotensin II plays a central role in amplifying Ly6C^hi^ monocytes and their precursors in the spleen red pulp of KP lung carcinoma-bearing mice as well as releasing monocytes from their splenic reservoir (28, 29). However, a conflicting view has been suggested in a different lung tumor model. During the development of Lewis lung carcinoma (LLC), the bone marrow primarily promotes monocyte production while the spleen plays a minor role in monocyte production (30). Monocytes produced from the bone marrow are more favored to migrate into and to be accumulated in the tumor region than those from the spleen (30). Although an increased accumulation of monocytes in the spleen is also detected in the LLC model, it is because the bone marrow primarily accelerates monocyte production and transfers the newly formed monocytes to the spleen; the spleen is not the primary source (30). As such, different context might have yielded the controversy on tumor monocyte development. Therefore, further studies need to be conducted in as many types of tumors as possible (31).

### Monocytes: Pro-tumoral vs. Anti-tumoral Functions in Solid Tumors

**Figure 1:**
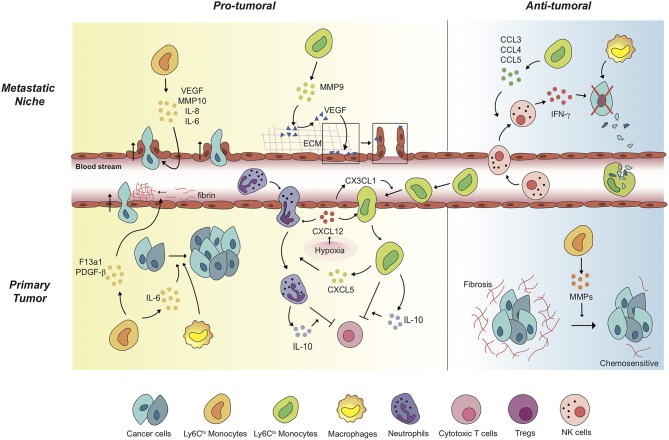
Monocytes mediate a variety of pro-tumoral and anti-tumoral mechanisms in a context-dependent way. In primary tumors, Ly6C^hi^ monocytes exert pro-tumoral effects to promote cancer cell proliferation and cancer cell intravasation. Of note, anti-angiogenic therapy induces Ly6C^lo^ monocyte-mediated immunosuppressive tumor microenvironment and triggers resistance against the therapy. Under the treatment, non-classical Ly6C^lo^ monocytes have been revealed to extravasate to primary tumor regions. The tumor-infiltrated Ly6C^lo^ monocytes significantly contribute to inhibition of cytotoxic T cell function. In metastatic niches, Ly6C^hi^ monocytes and Ly6C^lo^ monocytes facilitate cancer cell extravasation by secreting pro-angiogenic molecules and by mediating the release of ECM-bounded VEGF molecules. In contrast, these monocytes display anti-tumoral functions in different settings. In the lung metastatic sites, Ly6C^lo^ monocytes recruit tumor-killing NK cells, and scavenge tumor materials in the lung vasculature. Meanwhile, Ly6C^hi^ monocytes degrade fibrosis around cancer cells, which have the cancer cells acquire chemosensitivity upon treatments.

Other than the well-known feature as precursors of macrophage and dendritic cell populations, monocytes play a significant role *per se* in orchestrating the immune system not only in homeostatic condition (21), but also in tumor progression (7, 8, 32–35). Generally, high rate of monocyte infiltration into the tumor milieu indicates poor clinical prognosis of cancers (36, 37). Since each subset of monocytes has different functions in tumor progression depending on the context, it is momentous to decide which subset of monocytes should be targeted in each tumor. Distinct functions of Ly6C^hi^ monocytes and Ly6C^lo^ monocytes in solid tumors have been explored (Table 1; Figure 1). These monocytes play pro-tumoral or anti-tumoral roles, regulating diverse mechanisms ranging from angiogenesis to immune modulation in a context-dependent manner (Table 1; Figure 1).

**Table 1:** Context-derived heterogeneous functions of monocyte subsets.

**Type of monocytes**	**Function**		**Factor**	**Model**	**Cancer type/treatment**	**References**
Classical monocytes	Protumoral	Metastasis; Tumor cell extravasation	VEGFA	Mouse	MMTV-PyMT breast cancer	(32)
			VEGF, MMP-10, IL-8, TNF-α, PTGS2	Human	Renal cell carcinoma	(38)
		Metastasis; Cancer cell invasion	F13a1	Mouse	KLN205 lung squamous cell carcinoma	(39)
				Human	Lung cancer	
		Tumor fibrosis	PDGF-β	Mouse	Hepatocellular carcinoma	(40)
	Antitumoral	Degradation of tumor fibrosis	MMPs	Mouse	KPC pancreatic adenocarcinoma w/anti-CD40 treatment	(33)
Non-classical monocytes	Protumoral	Immunosuppression	CXCL5, IL-10	Mouse	CT26, SL4 colorectal cancer w/anti-VEGFR2 therapy	(7, 8)
		Angiogenesis	MMP-9	Human cancer cell xenograft	DLD1, HCT116 human colorectal carcinoma	(41)
	Antitumoral	NK cell recruitment	CCL3, CCL4, CCL5	Mouse, Human cancer cell xenograft, Human	B16F10 melanoma, A375 human melanoma, MMTV-PyMT breast cancer, Human lung cancer specimen (early stage)	(35, 42, 43)
		NK cell activation	IL-15	Mouse	B16F10, B16F0 melanoma	(44)

*Summarizes diverse protumoral and antitumoral functions of monocyte subsets (classical and non-classical monocytes) and their related factors in each model*.

#### Recruitment of Classical Monocytes and Their Functions in Solid Tumors

Ly6C^hi^ classical monocytes have been mostly reported to play pro-tumoral functions once recruited to the tumor microenvironment (Table 1; Figure 1). Ly6C^hi^ monocytes express high levels of CCR2 on their surface (32). CCR2 mediates the migration of Ly6C^hi^ monocytes from the bone marrow to CCL2-secreting tumor milieu in PyMT spontaneous breast carcinoma, KCKO pancreatic carcinoma, and MC38 colorectal carcinoma (29, 45, 46). These recruited classical monocytes release VEGFA (a major stimulator of angiogenesis) to facilitate tumor cell extravasation and lung metastasis (32, 47). In human pancreatic tumor as well as murine pancreatic lesion model, the tumor microenvironment releases CCL2 and thereby actively recruits CCR2-expressing CD14^+^CD16^−^ classical monocytes from bone marrow to blood stream, which is a prognostic factor of worse outcome (45). In contrast, CCR2 inhibition attenuates the mobilization and thus leads to forming an anti-tumoral immune environment in KCKO pancreatic carcinoma and MC38 colorectal carcinoma (45, 46). In human RCC patients and xenograft models, the IL-1β/IL-1R interaction activates the MyD88-NF-kB signaling pathway, and thereby enables classical monocytes with pro-tumoral phenotypes to upregulate pro-tumoral genes such as VEGF, MMP-10, IL-8, TNF-α, and PTGS2 (38). Ly6C^hi^ monocytes/CD14^+^CD16^−^ monocytes also facilitate cancer cell invasion and metastases via expressing F13a1 to promote fibrin cross-linking not only in murine KLN205 lung squamous cell carcinoma but also in human lung cancer, implicating poor survivals (39). As such, in hepatocellular carcinoma (HCC), Gr-1+ myeloid cells which contain Ly6C^hi^ monocyte population play pro-tumoral function supporting tumor fibrosis by secreting platelet-derived growth factor-beta (PDGF-β), a pro-fibrotic growth factor (40).

Moreover, classical monocytes play a major role in establishing a cancer therapy-resistant microenvironment (34, 48, 49). Doxorubicin treatment on MMTV-PyMT breast carcinoma, for induction of necrotic cell death, triggers the enhanced infiltration of CCR2-expressing monocytes. At later stages of cancer, this backfires; these monocytes have been revealed responsible for resistance against doxorubicin, promoting tumor relapse after treatment (48). In 4T1 and MMTV-PyMT breast carcinoma, paclitaxel treatment induces the secretion of tumor-derived extracellular vesicles (EVs), and these EVs upregulate pulmonary CCL2 expression to elicit classical monocyte expansion establishing a lung pre-metastatic niche (34). Applying radiotherapy on KPC pancreatic carcinoma also leads to a significant increase in CCL2 production by tumor cells. Subsequent recruitment of classical monocytes thereby endows the tumor with resistance against the cancer treatment (49). Use of anti-CCL2 antibodies selectively restrains radiotherapy-dependent recruitment of classical monocytes, impeding tumor progression when combined with radiotherapy (49).

Based on these findings, treatment with anti-CCL2 antibody might sound attractive for tumor regression. However, the following study has proposed a caution for anti-CCL2 mono-treatment. During anti-CCL2 treatment in 4T1, J110, and Met-1 mammary carcinoma, a large population of the classical monocytes is retained within the bloodstream, and their homing to the primary tumor or to the metastatic site is attenuated (50). However, after anti-CCL2 treatment cessation, monocytes initiate their migration to the lungs, and the level of IL-6 rises within the lungs. The increased level of IL-6 augments pro-angiogenic VEGF-A expression in classical monocytes, and thereby accelerates tumor metastasis (50). IL-6RA is largely expressed in Ly6C^hi^ monocytes, and anti-IL-6R antibodies effectively target Ly6C^hi^ monocytes (51, 52). Notably, IL-6-IL-6R interaction not only promotes VEGF-A secretion from classical monocytes but also activates the STAT3 signaling pathway in cancer cells, which enhances tumor cell proliferation in pancreatic ductal adenocarcinoma (PDAC) (50–53). IL-6 is also strongly induced in adipocytes and tumor-infiltrated myeloid cells after anti-VEGF treatment on overweight breast cancer patients. The upregulated IL-6 mediates resistance to anti-VEGF therapy, leading to the proliferation of cancer cells and dysfunctional angiogenesis (54). IL-6 inhibition increases the tumor microenvironment's sensitivity to chemotherapy and anti-angiogenic therapy and promotes tumor cell death (52, 54).

Contrary to the pro-tumoral properties of Ly6C^hi^/CD14^+^CD16^−^ monocytes explicated above, it has also been reported that these classical monocytes play anti-tumoral functions in certain treatments (Table 1; Figure 1). Tumor fibrosis promotes tumor progression by increasing collagen deposition, reducing T cell infiltration, and inducing pro-tumoral macrophage polarization (33, 55–58). A distinct class of Mac1^+^F4/80^−^Msr1^+^ceacam1^+^Ly6C^lo^ monocytes has been recently discovered to promote fibrosis in C/EBPβ dependent manner (59). Meanwhile, Ly6C^hi^ monocyte infiltration into KPC pancreatic adenocarcinoma via IFN-γ and CCL2 following anti-CD40 treatment has been reported to facilitate degradation of tumor fibrosis, increasing the efficacy of the chemotherapy on PDAC while Ly6C^hi^ monocyte-containing Gr-1^+^ myeloid cells in HCC play pro-fibrotic roles (33, 40, 55).

#### Recruitment of Non-classical Monocytes and Their Functions in Solid Tumors

On the other hand, Ly6C^lo^/CD14^−^CD16^+^ non-classical monocytes have independent mechanisms for infiltration to tumors, and their functions are context-dependent. In models of colorectal cancer, Jung et al. have firstly revealed immunosuppressive functions of non-classical monocytes in any context, including cancers (7, 8). Anti-angiogenic therapy leads to non-classical monocyte influx to CX_3_CL1-secreting tumor milieu. Then these recruited non-classical monocytes secrete CXCL5, and mediate a massive infiltration of CXCR2-expressing neutrophils through the highly specific chemokine axis (7). This finding echoes a previous finding also showing that non-classical monocytes recruit neutrophils, albeit mediated by CXCL1—not CXCL5—in a different disease condition outside oncology (60). These tumor-infiltrating non-classical monocytes and neutrophils release immunosuppressive cytokines including IL-10 which inhibits infiltration and activity of cytotoxic T lymphocytes in tumors (7, 8) (Table 1; Figure 1). Jung et al. also successfully developed several therapeutic strategies targeting these non-classical monocyte-mediated cascades by blocking their infiltration and activity (7, 8). Through a series of *in silico* and *in vitro* screening, novel siRNA sequences against CX_3_CL1 with potent knock-down efficacy were identified. The siRNA was formulated with nanoparticles particularly designed for endothelial cell-specific delivery, which resulted in inhibiting Ly6C^lo^ monocyte infiltration and subsequently reduced tumor growth (7). Notably, CXCR4 was discovered to be a critical chemokine receptor expressed on non-classical monocytes and neutrophils (8). CXCL12/CXCR4 axis in these cells mediates restrained cytotoxic T cell infiltration and builds up immunosuppressive tumor microenvironment in CT26, SL4 colorectal carcinoma, and E0771, MCa-M3C mammary carcinoma (8, 61). Supporting this finding, AMD3100 which is a potent CXCR4 inhibitor, also known as plerixafor, efficiently hinders the recruitment of non-classical monocytes, improving the treatment efficacy of anti-VEGFR2 therapy. This suggests the potential of rapid clinical translation, since AMD3100 is already an FDA-approved CXCR4 blocker being used in the clinic for other uses (8, 61).

Despite the several pro-tumoral features of Ly6C^lo^/CD14^−^CD16^+^ non-classical monocytes, these monocytes also display anti-tumoral properties in different tumor/treatment conditions (Table 1; Figure 1). In B16F10 melanoma and MMTV-PyMT spontaneous mammary carcinoma, non-classical monocytes play a pivotal role in engulfing tumor material in the lung and attenuating tumor metastasis and activating NK cells (17, 35). In B16F10 and B16F0 melanoma, non-classical monocytes also activate NK cells by releasing IL-15, which is a determinant cytokine for NK cells' homeostasis, activation and effector function, preventing lung metastases in primary tumor-bearing mice (44). In B16F10 melanoma and A375 human melanoma xenograft models, exosomes secreted from non-metastatic cancer cells promoted the expansion of non-classical monocytes in the bone marrow (42). The expanded population of the non-classical monocytes leads to recruiting NK cells which function in cancer cell clearance at the pre-metastatic niche (42). This NK cell-recruiting function of non-classical monocytes have been reconfirmed in early stage lung cancer patients (43). Based on these findings, reduced CD16^+^ non-classical monocytes might be correlated with NK cell paucity in this lung tumor lesions (43). According to *ex vivo* study of patients with stage IV cutaneous melanoma, CD14^−^CD16^+^ non-classical monocytes kill regulatory T lymphocytes (Tregs) by assisting ipilimumab, anti-cytotoxic T lymphocyte associated antigen 4 (CTLA4) monoclonal antibody, -mediated ADCC (antibody-dependent cell-mediated cytotoxicity) (62).

Importantly, it had been widely believed that non-classical monocytes are not able to extravasate out of blood vessels. Instead, they were known to stay inside vasculature and patrol the endothelium, which gave these monocytes the nickname “patrolling monocytes” (9). However, recent studies strongly suggest that they do have the capability of transmigration and actively infiltrate into tissues, proven by state-of-the-art *in vivo* imaging techniques (7, 8). Supporting this, in DLD1 and HCT116 human colorectal carcinoma, recruited human patrolling monocytes in tumors secrete matrix metalloproteinase 9 (MMP9), a proteolytic enzyme fostering angiogenesis, triggering a release of matrix-bound VEGFA. This accelerates the extravasation and accumulation of these pro-angiogenic patrolling monocytes, promoting tumor progression (41). This also validates the first finding of non-classical monocyte extravasation directly visualized by intravital microscopic imaging (7, 8).

#### Tie2-Expressing Monocytes

Other than the traditional classification of monocytes by Ly6C expression level, another classification method by Tie2 (angiopoietin receptor) expression exists. Tie2-expressing monocytes (TEMs) are a monocyte population present in both human and mouse peripheral blood and tumor, and are localized in perivascular spaces but not incorporated with vascular endothelial cells (63, 64). Angiopoietin-1 (Ang-1), a Tie2 ligand, is likely to promote the recruitment of TEMs to tumor vasculature before the turn-on of the angiogenic switch in early stages of N202 breast carcinoma, Rip1-Tag2 pancreatic insulinoma and U87 human glioma (63, 65, 66). In a following study, it was also elucidated that Angiopoietin-2 (Ang-2), another Tie2 ligand upregulated in tumor hypoxia, can also recruit TEMs. The TEMs are then reprogrammed to show proangiogenic phenotypes (67, 68). Meanwhile, Collagen triple-helix repeat-containing 1 (CTHRC1) secreted by several malignant tumors has been reported to recruit TEMs to the tumor microenvironment through upregulation of Ang-2 in endothelial cells and promote metastasis in human MiaPaCa-2, CFPAC-1, and Panc-1 pancreatic cancers (69). Recruited TEMs promote angiogenesis via secretion of a proangiogenic molecule, basic fibroblast growth factor (bFGF) (63–66). Also, Ang-2 and hypoxia cause TEM influx into the tumor microenvironment, and the TEMs mediate downregulation of TNF-α supporting cancer cell survival and causing metastasis of the primary tumor (63, 67, 70). Blockade of Ang-2 impedes tumor angiogenesis in MMTV-PyMT breast carcinoma and Rip1-Tag2 pancreatic insulinoma through downregulation of Tie2 in TEMs (71).

## Neutrophils: From Development to Deployment

### Neutrophil Development

Neutrophils are another myeloid compartment which plays critical roles both in homeostatic condition and tumor context. There is a train of precursors to be passed through to generate mature neutrophils in the bone marrow (72). Hematopoietic stem cells (HSCs) give rise to multipotent progenitors (MPPs), lymphoid primed multipotent progenitors (LMPPs), and granulocyte/macrophage progenitors (GMPs) in this very order (72). There are several more stages to go to be differentiated to neutrophils, namely a series of myeloblasts, promyelocytes, myelocytes, metamyelocytes, band cells, and finally neutrophils (72). These steps for neutrophil generation occur under major regulation by the granulocyte-colony stimulating factor (G-CSF), granulocyte–macrophage-colony stimulating factor (GM-CSF) and also minor regulation by other molecules such as IL-6 and KIT ligand (KITL) (73). Differentiating neutrophils express the G-CSF receptor (G-CSFR) throughout the myeloid lineage (73). During development in the bone marrow, neutrophils acquire three types of granules sequentially; azurophil (primary) granules which retain myeloperoxidase regulated by transcription factors C/EBPα and Gfi-1, specific (secondary) granules which contain lactoferrin mostly regulated by C/EBPε, and gelatinase (tertiary) granules which contain MMP9 regulated by C/EBPβ, C/EBPδ, C/EBPγ, and PU.1 (74, 75). Of note, mass cytometry has recently found new proliferative precursors of neutrophils after GMP stage which further differentiate to immature neutrophils and mature neutrophils with regulation of C/EBPε (76). Although the bone marrow is primarily responsible for the neutrophil formation, the spleen can be an alternative source of neutrophils during emergency granulopoiesis derived from cancer progression (73). In KP lung adenocarcinoma, splenic hematopoietic stem cells, and progenitor cells produce neutrophils during tumor progression (27). Presence of cancer cells upregulates the expression of several factors accelerating neutrophil development. The expression of CXCL1, CXCL2, CXCL5, and CXCL8, which are CXCR2 ligands, and the expression of KITL and GM-CSF are strongly enhanced by KRAS signaling in cancer cells and tumor-derived hypoxia (73). Moreover, IL-1β-producing macrophages and IL-17-producing γδ T cells secrete G-CSF to promote neutrophil development in the tumor (73). Cancer cells accelerate secretion of these cytokines and chemokines to instigate overactive granulopoiesis and neutrophilia (73). The accelerated secretion of these factors promotes the release of immature neutrophils to the blood stream, resulting in an increased number of circulating neutrophils (73). In 4T07, 4T1 mammary carcinoma, LLC, and Kras-driven pancreatic carcinoma, G-CSF production is also facilitated via RAS/MEK/ERK pathway in cancer cells, promoting recruitment of neutrophils (77). Meanwhile, type I IFNs from tumor trigger differentiation of neutrophils to achieve an anti-tumoral phenotype, reducing not only CXCR4 expression in neutrophils which mediates tumor-homing, but also VEGF and MMP9 expression (78, 79). Moreover, type I IFNs suppress G-CSF signaling pathways in neutrophils, thereby reducing expression of Bv8, S100A8, S100A9, and MMP9 so that they can attenuate the formation of the pre-metastatic niche (78, 79). Inhibition of type I IFNs impairs cytotoxicity of neutrophils and promotes metastasis of B16F10 melanoma, MCA205 fibrosarcoma, 4T1 mammary carcinoma and LLC mediated by neutrophils (78, 79).

### Neutrophils: Pro-tumoral vs. Anti-tumoral Functions in Solid Tumors

**Figure 2:**
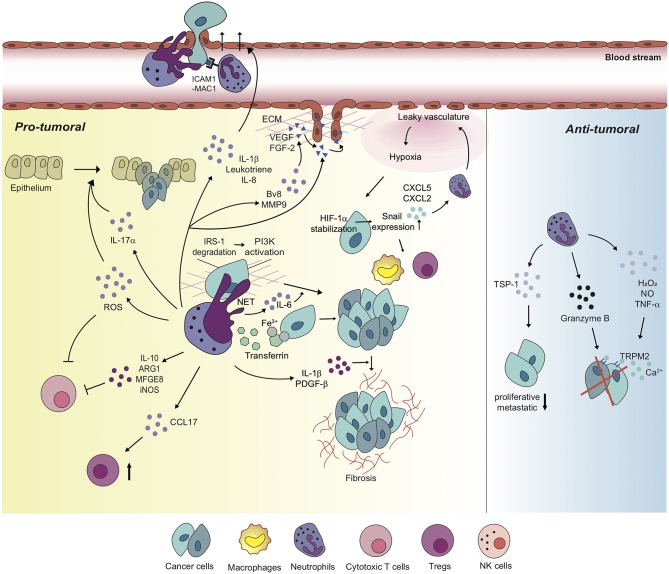
Neutrophils differentially regulate tumor microenvironment with diverse mechanisms. Neutrophils perform pro-tumoral roles in most tumor settings, promoting tumorigenesis, and cancer cell proliferation via diverse mechanisms. Moreover, neutrophils regulate the functions of other immune cells including cytotoxic T cells and Tregs in order to build up tumor-favorable tumor microenvironment. On the one hand, neutrophils stimulate tumor angiogenesis via inducing the release of VEGF and FGF-2 from ECM or secreting pro-angiogenic molecules, themselves. Furthermore, metastatic competence of cancer cells can be achieved by physical interaction with neutrophils and neutrophil-derived secretory molecules, facilitated to extravasate to secondary tumor sites. Neutrophils also create a positive feedback loop with cancer cells toward the formation of the tumor-supportive microenvironment, developing dysfunctional vasculature around the tumor, leading to hypoxia which recruits more neutrophils and pro-tumoral immune cells into tumor milieu. On the other hand, a couple of studies indicate anti-tumoral functions of neutrophils in different contexts. In these contexts, neutrophils perform a cytotoxic function on cancer cells, and have cancer cells lose proliferative and metastatic properties.

Functions of neutrophils in the tumor microenvironment vary by context including types of tumor, stages of tumor progression, and different therapies (Table 2; Figure 2).

**Table 2:** Context-dependent multifaceted functions of neutrophils.

	**Function**		**Factor**	**Model**	**Cancer type/treatment**	**References**
Neutrophils	Protumoral	Tumor initiation	Neutrophil elastase	Mouse	Kras mutant	(80)
			ROS, RNS	Mouse	Colon cancer	(81)
		Cancer cell proliferation	NETs (Neutrophil elastase traps), HMGB-1	Mouse	MC38 colorectal cancer w/ischemia and reperfusion injury	(82)
			Neutrophil elastase	Mouse	A549 lung adenocarcinoma	(83)
			IL-6, IL-1β	Mouse	4T1 breast cancer	(84)
			Transferrin	Mouse	4T1 breast cancer	(85)
		Cancer cell colonization; Differentiation from monocytes to fibrocytes	MMP-9	Mouse	CMT93 colon carcinoma	(86, 87)
		Fibrosis	MAP kinase pathway	Mouse	HCA-1 hepatocellular carcinoma w/Sorafenib treatment	(88)
			IL-1β	Mouse	AK4.4, Pan02, KPC, iKRAS pancreatic adenocarcinoma	(58)
		Macrophage recruitment	MAP kinase pathway	Mouse	TRAMP-C1 prostate cancer, E0771 breast cancer w/VEGF blockade	(89)
		T cell suppression	IL-10	Mouse, Human cancer cell xenograft	CT26, SL4 colorectal cancer w/anti-VEGFR2 therapy/LS174T human colorectal cancer	(7, 8, 90)
			PD-L1	Mouse	H22-generated hepatoma	(91)
			IL-10, LGALS9, ARG1, MFGE8	Mouse	KP lung carcinoma	(92)
			Nos2	Mouse	KEP breast carcinoma, AB12 mesothelioma, LKRM lung carcinoma, LLC	(93, 94)
			ARG1	Human	Non-small cell lung cancer	(95)
		Regulatory T cell attraction	CCL17	Mouse	LLC, AB12 mesothelioma	(96)
		Angiogenesis	Bv8	Mouse	Rip-Tag pancreatic insulinoma	(97, 98)
			MMP-9	Mouse, Human cancer cell xenograft	Rip1-Tag2 pancreatic insulinoma, L929 fibrosarcoma, B16-F10 melanoma, LLC, HPV-	(99–102)
					15-induced squamouse carcinoma, HT-1080 fibrosarcoma/PC-3 human prostate carcinoma	
			VEGF	Human	Oral cavity cancer	(103)
			FGF2	Mouse, Human cancer cell xenograft	Pan02, KPC pancreatic carcinoma/HT29, HCT-116, Lovo human colon cancer	(104)
		Metastasis; Tumor cell extravasation	IL-1β, Leukotriene, IL-8	Mouse, Human cancer cell xenograft	4T1, D2A1 breast cancer/Human MDA-MB-	(105–107)
					231 breast cancer, human A375-MA2, WM35, C8161.C19, UACC903 melanoma	
		Metastasis; Epithelial-mesenchymal transition (EMT)	IL-17α	*In vitro* human cancer cell	(*In vitro*) Human MKN45, MKN74 gastric cancer	(108)
		Metastasis; Bridge between ICAM-1-expressing cancer cells and endothelial cells	MAC-1	Mouse, Human cancer cell xenograft	H50 Lewis Lung carcinoma/Human A549 lung carcinoma	(109)
		Cancer cell retention	NETs (Neutrophil elastase traps)	Mouse	H59 Lewis lung carcinoma w/cecal ligation and puncture/MC38 colorectal cancer w/ischemia and reperfusion injury	(82, 110)
		Activation of dormant cancer cell		Mouse	D2.0R breast cancer	(111)
	Antitumoral	Tumor cell death	TNF-α, NO, H_2_O_2_	Mouse	LLC, AB12 mesothelioma	(112)
			Granzyme B		CT26 colon cancer	(113)
			H_2_O_2_		AT3, 4T1, MMTV-PyMT breast cancer	(114, 115)
		IL-17+ γδ T cell suppression	ROS	Mouse	B16F10 melanoma, Hepa1-6 hepatoma	(116)
		Impairment of tumor cell proliferation	Tsp-1	Human cancer cell xenograft	Human PC3 prostate cancer, human MDA- MB-231 breast cancer	(117)
		Stimulation of T cell response	CD54, CD86, OX40L, and 4-1BBL	Human	Lung cancer (early stage)	(118)

*Summarizes multiple protumoral and antitumoral functions of neutrophils and their responsible factors in each different context*.

#### Recruitment of Neutrophils to Tumor Milieu

As mentioned above, tumors promote the early release of neutrophils yet with immature phenotypes from the bone marrow. There are several ligand-receptor axes studied for neutrophil recruitment into the tumor. Upon research on diverse tumor models, it has been revealed that CXCR2 is a pressing chemokine receptor which recruits neutrophils to the tumor (7, 8, 119, 120). In KPC pancreatic carcinoma and inflammation-driven and spontaneous intestinal adenocarcinoma, the migration of myeloid cells, especially neutrophils, to the tumor microenvironment is impaired when CXCR2 signaling is suppressed (119, 120). This enhances tumor cell apoptosis and restrains tumorigenesis, resulting in a failure to set up a metastatic niche (119, 120). Moreover, activated neutrophils also express CCR7 on their membrane, which pushes those cells to tumor sites in response to CCL19, CCL21, and GM-CSF secretion (121). IL-17 also triggers neutrophil recruitment to tumor sites in 4T1 breast carcinoma (122), KRAS mutated lung carcinoma (123), and ovarian carcinoma (124). The recruited neutrophils present high expression of tumor-promoting genes such as TNF-α, CXCL1, MMP9, and VEGF (122). In zebrafish larvae model of glioblastoma initiation, neutrophils are actively recruited to KRAS-transformed cells very early in oncogenesis via the CXCL8-CXCR1 signaling axis, and this recruitment contributes to the proliferation of tumor-initiating cells (125). Gastrin-releasing peptide (GRP)-GRP receptor (GRPR) axis can also induce neutrophil migration in the tumor (126, 127). In A375 and M24met human melanoma, CXCL5 overexpression by tumor cells enhances neutrophil recruitment and infiltration into primary tumors and tumor lymphatic vessels (128). It triggers the proximal interaction between neutrophils and cancer cells near the lymphatic endothelial cells in order to help trans-endothelial migration of the cancer cells (128). In SL4 and CT26 colorectal cancer, tumor-infiltrated Ly6C^lo^ monocytes induced by anti-VEGF therapy can also recruit CXCR2-expressing neutrophils to the tumor site via the CXCL5-CXCR2 and CXCL12-CXCR4 axes (7, 8). Albeit in a different disease setting, it has been also reported that monocytes recruit neutrophils in a TLR7-dependent manner through CXCL1 (60), different from the case of cancer context where non-classical monocytes-derived CXCL5 was newly discovered as the key chemokine attracting neutrophils (7, 8).

#### Pro-tumoral Functions of Neutrophils in Solid Tumors

Recruited neutrophils from the blood stream have potent influences on various components of tumor progression and metastasis, including tumor initiation, cancer cell survival/proliferation, immune modulation, angiogenesis, and intra/extravasation of cancer cells (Table 2; Figure 2).

##### Neutrophils and tumor initiation

In Kras mutant mice, airway inflammation induces secretion of IL-8 by lung keratinocytes, recruiting CXCR2-expressing neutrophils to the inflammation site (80). Neutrophil elastase (NE), a powerful serine protease exclusively found in primary granules of neutrophils, facilitates inflammation-mediated tumor initiation in the lung (80). H. hepaicus-induced colitis triggers tissue infiltration of MPO^+^ neutrophils and macrophages into the infected sites (81). These neutrophils and macrophages generate reactive oxygen species (ROS) and reactive nitrogen species (RNS) which subsequently cause molecular damage, promoting tumorigenesis (81). Transcriptional analysis reveals that genes involved in repairing DNA damage were downregulated, but genes associated with reactive chemical species generation were upregulated in infected colons (with no impact on cell proliferation) (81).

##### Neutrophils and cancer cell survival/proliferation

Beyond tumorigenic functions of neutrophils, their roles in cancer cell survival and proliferation have been also elucidated well. Overexpression of insulin receptor substrate 1 (IRS-1) is known to reduce tumor growth (83). In A549 lung adenocarcinoma, neutrophil elastase (NE) degrades IRS-1 in cancer cells, thereby causing tumor cell proliferation. PI3K signaling pathway alteration mediates this process by fostering the interaction with PDGF-receptor (83). In 4T1 breast carcinoma, transferrin, an iron-transporting protein secreted by neutrophils, binds to its receptor expressed on cancer cells (85). Then the transferrin supplies iron to the cancer cells for their proliferation (85). As tumor growth and metastasis are promoted, transferrin secretion by neutrophils increases (85). Sorafenib, a rapidly accelerated fibrosarcoma (RAF) inhibitor, is commonly used as treatment for HCC. However, the use of sorafenib causes side effects and resistance such as tumor desmoplasia. Gr-1^+^ myeloid cells including neutrophils have been revealed to be responsible for the resistance (40, 88, 129, 130). Sorafenib treatment induces tumor hypoxia, which upregulates CXCL12 expression in HCA-1 cancer cells and stromal cells. Then, CXCR4-expressing Gr-1^+^ myeloid cells are promoted to infiltrate to CXCL12-secreting tumor sites, and the infiltrated cells support differentiation and activation of hepatic stellate cells via the MAP kinase pathway and fibrosis in HCC (40). Of note, CXCL12-CXCR4 axis triggers increased infiltration of Tregs and M2 type macrophages and upregulation of intratumoral PD-L1 in HCA-1 HCC (88). Moreover, in Ak4.4, Pan02, KPC, and iKRAS pancreatic adenocarcinoma, adipocytes of obese population secrete increased levels of IL-1β to recruit neutrophils to the tumor along with enhancing Treg infiltration, and hindrance of CD8^+^ T cell infiltration (58). The recruited neutrophils then activate pancreatic stellate cells via IL-1β secretion to accelerate fibrosis, which promotes tumor growth and reduces sensitivity to chemotherapy (58).

##### Neutrophils and immune modulation

Neutrophils play essential roles in tumor growth and metastasis not only to regulate cancer cell proliferation and survival, but also to modulate innate and adaptive immunity. The recruited neutrophils via CXCL12-CXCR4 axis secrete IL-10 that suppresses cytotoxic T cell function on tumor cells, which then causes anti-VEGF therapy resistance in SL4 and CT26 colorectal carcinoma (7, 8). Similar findings have been recently reported in LS174T human colorectal carcinoma (90), which confirms the previous observations in preclinical murine models (7, 8). The CXCL12-CXCR4 axis in myeloid cells including neutrophils is also responsible for NK cell apoptosis and inactivation by enhancing the Fas signaling pathway and restraining IL-18 production in neutrophils, respectively, in metastatic B16F0 melanoma, PyMT breast carcinoma, and YAC-1 lymphoma (131). Neutrophil-mediated modulation of NK cells has been confirmed in 4T1 and D2A1 metastatic mammary carcinoma context (D2A1 inoculation after 4T1 injection) as well (105). The expanded population of neutrophils near metastatic sites inhibits functional activation of NK cells, and thus the NK cells lose their ability to clear intraluminal tumor cells (105). This consequently comes across with a favorable environment for cancer cell survival and metastasis (105). CXCR4 depletion in those myeloid cells recovers the tumor-killing capacity of NK cells (131). In H22-generated hepatoma-bearing mice, PD-L1 is upregulated in tumor-infiltrating neutrophils (TINs), induced by GM-CSF and TNF-α secretion from the tumor microenvironment (91). The overexpressed PD-L1 of neutrophils suppresses proliferation and activation of PD-1^+^ T cells, dampening anti-tumor immunity (91). In STK11/LKB1-deficient KP lung carcinoma, recruited neutrophils produce suppressive factors such as IL-10, LGALS9, Arginase1 (ARG1), and Milk fat globulin EGF factor (MFGE8) which are also involved in cytotoxic T cell suppression as well as the tumor-promoting cytokine IL-6 (92). In KEP breast carcinoma, Nos2, the gene encoding iNOS, is largely upregulated in neutrophils (93). Then the neutrophils suppress CD8^+^ T cell activity via NO from iNOS, promoting lung metastasis (93). Effect of neutrophil NO production on CD8^+^ T cell apoptosis has been also confirmed in AB12 mesothelioma, LKRM lung carcinoma, and LLC (94). TNFα-mediated iNOS upregulation and NO secretion in neutrophils induce the apoptosis of non-activated CD8^+^ T cells via direct contact between cells in these tumor contexts (94). ARG I secretion by neutrophils has been also uncovered to affect T cell suppression by degrading extracellular arginine in non-small cell lung carcinoma patients (95). IL-8 and TNF-α secretions are enhanced in non-small cell lung carcinoma patients, and these cytokines induce ARG I release from exocytosis of granules in neutrophils (95). In 4T1 mammary carcinoma, SCF-c-kit signaling increases c-kit+ neutrophil frequency in the circulatory system. Even in nutrient-limited tumor microenvironments, these neutrophils exploit fatty acid metabolism to maintain mitochondrial function and support ROS production, resulting in T cell suppression (132). This also echoes the formal observations of immunosuppressive neutrophils in colon cancers (7, 8). Meanwhile, a research adopting mass cytometry and single-cell RNA sequencing has recently revealed that a unipotent precursor of neutrophils promotes B16F10 melanoma progression via inhibition of pro-inflammatory T cell activation, eliciting an immunosuppressive microenvironment around the tumor (133).

Neutrophils affect tumor progression by regulating other immune cells beyond NK cells and cytotoxic T cells. In LLC and AB12 mesothelioma, neutrophils attract Tregs via CCL17 secretion and thus scupper the formation of an anti-tumor immune microenvironment (96). Furthermore, blockade of VEGF in TRAMP-C1 prostate carcinoma and E0771 breast carcinoma triggers Gr-1^+^ myeloid cell recruitment which mediates macrophage recruitment to the tumor microenvironment via activation of p38 mitogen-activated protein kinase (MAPK) to promote lung metastasis (89).

Myeloid-derived suppressor cells (MDSCs) are immunophenotypically defined as CD11b^+^ Gr-1^+^ cells (in mice) and possess pro-tumorigenic functions including immune suppression (134). These MDSCs can be further classified into granulocytic (or polymorphonuclear) MDSCs [gMDSCs or PMN-MDSCs] (CD11b^+^ Ly6C^−^ Ly6G^+^ in mouse, CD11b^+^ CD14^−^ CD15^+^ in human) and monocytic MDSCs [mMDSCs] (CD11b^+^ Ly6C^+^ Ly6G^−^ in mouse, CD11b^+^ CD14^+^ HLA-DR^low^ CD15^−^ in human) (134). Although we do not doubt that MDSCs play important roles in regulating tumor progression, the definition of MDSCs is still under debate since it is difficult to clearly discriminate the heterogeneous myeloid cell mixtures only with markers being currently used (7, 8). Indeed, phenotypical and functional features of MDSCs are considerably overlapped with those of monocytes and neutrophils, which we discuss in depth throughout this review. Therefore, we would rather not go into details of MDSCs here.

##### Neutrophils and angiogenesis

Even though cancer cells and cancer-associated fibroblasts are considerably responsible for the source of angiogenic factors, tumor-infiltrated myeloid cells including neutrophils also exert potent properties in tumor angiogenesis over diverse tumor settings (135). Upregulation of Bv8 following STAT3 activation is responsible for neutrophil-mediated tumor angiogenesis in the early stages of Rip-Tag pancreatic insulinoma (97, 98). In Rip1-Tag2 pancreatic insulinoma, L929 fibrosarcoma, B16-F10 melanoma, LLC, HPV-15-induced squamous carcinoma, HT-1080 fibrosarcoma, and PC-3 human prostate carcinoma, neutrophils infiltrated to the tumors majorly secrete MMP9 remodeling the ECM to release VEGF and FGF-2, and activating them to trigger chronic angiogenesis and thereby promotes tumor progression (99–102, 135). Meanwhile, in oral cavity cancer patients, neutrophils actively secrete VEGF, and promote tumor angiogenesis and metastasis (103). By studying HT29, HCT-116, LoVo human colon carcinoma and Pan02, KPC murine pancreatic carcinoma, it has also been studied that neutrophils are the main source of FGF2 (104). Here, these neutrophils play a proangiogenic role to develop unsystematic tumor vasculature and prompt liver metastasis, facilitating endothelial cell proliferation and migration (104). Hence, inhibition of FGF2 delays tumor growth via normalizing the vasculature (104). In KP lung carcinoma, neutrophils alter angiogenesis around tumor tissue, causing a hypoxic environment (136). HIF1α stabilization induced by hypoxia increases expression of the Snail gene in cancer cells (136). The Snail-expressing cancer cells secrete increased levels of CXCL5 and CXCL2 to recruit more pro-tumoral neutrophils to the tumor, creating a positive amplifying loop to facilitate tumor growth (136, 137). Notably, it has been revealed that Snail has a pro-tumorigenic influence via recruiting pro-tumoral M2 macrophages as well in 4T1 breast cancer and LLC1 lung cancer (137). Snail also induces Treg differentiation and impairs the activity of dendritic cells in B16F10 melanoma (138).

##### Neutrophils and metastasis

While affecting primary tumor cell proliferation, modulation of the immune microenvironment, and angiogenesis, neutrophils also play significant roles in supervising tumor metastasis. It has been reported that neutrophil infiltration is essential to endow non-malignant BMT-11 fibrosarcoma cancer cells with malignant and metastatic phenotypes (139). The metastatic incidence is significantly reduced with the anti-Gr1 antibody-mediated neutrophil depletion in blood circulation or integrin β2 knockout mice lacking in neutrophil extravasation (139). Of note, integrin β2 mediates neutrophil adhesion on activated endothelium with high affinity, which leads to transmigration of neutrophils across the endothelium (140, 141). A number of subsequent researches have endeavored to illuminate diverse factors and mechanisms which can explain functions of neutrophils on tumor metastasis. In 4T1 and D2A1 metastatic mammary carcinoma (D2A1 injection after 4T1 injection), recruited neutrophils activate endothelial cells via secretion of IL-1β. This, in turn, facilitates trans-endothelial migration of intraluminal tumor cells, forming small protrusions of the cell bodies across the endothelial layer (105, 106). In 4T1 breast carcinoma and human MDA-MB-231 breast carcinoma, leukotrienes derived from neutrophils transform cancer cell populations to acquire highly metastatic competence in lung pre-metastatic sites (106). The metastatic competence of cancer cells can be also acquired by IL-17α secretion of neutrophils in gastric cancer (108). Of note, epithelial-mesenchymal transition (EMT) endows cancer cells with invasive properties and high-grade malignancy (108, 142). Coculture of gastric cancer patient-derived neutrophils and human MKN45, MKN74 gastric cancer cells has proven that IL-17α activates JAK2/STAT3 axis in the cancer cells following by their acquisition of mesenchymal characteristics, and the IL-17α is mostly derived from tumor-associated neutrophils (TANs) (108).

Employment of a multiplexed microfluidic model of the human microvasculature has revealed that neutrophils also secrete IL-8 by themselves (143). The self-secreted IL-8 induces not only neutrophil sequestration in A375-MA2 human melanoma cells but the interference of endothelial barrier function, supporting cancer cell extravasation (143). In human WM35, A375, C8161.C19, and UACC903 melanoma, IL-8 secreted from entrapped melanoma cells attracts neutrophils and increases integrin β2, specifically MAC-1, on the neutrophils. This leads to the enhancement of neutrophil-melanoma cell interaction, facilitating lung metastasis (107). There is another research subsequently conducted which confirms the interaction between MAC-1 and ICAM1 (neutrophils and cancer cells, respectively) (109). In H59 Lewis lung carcinoma and A549 human lung carcinoma, MAC-1 on neutrophils acts as a bridge between ICAM-1-expressing cancer cells and endothelial cells in favor of liver metastasis (109). Even though both pieces of research by Huh et al. (107) and Spicer et al. (109) have elucidated the MAC-1-ICAM-1 interaction and highlighted the significant function of neutrophils on tumor metastasis, the finding of Spicer et al. (109), is incompatible with Huh et al. (107), in that neutrophils come first to metastatic sites, and then circulating tumor cells directly adhere to the arrested neutrophils in the early step of metastasis. Interaction between neutrophils and circulating tumor cells in the bloodstream has also been elucidated to be mediated via VCAM-1 in 4T1 breast carcinoma (84). Also, the neutrophils physically clustered with circulating 4T1 breast cancer cells support the cancer cell cycle progression, secreting IL-6 and IL-1β, and promote metastasis of cancer cells (84). In MCF-7 human ER+ breast cancer, estradiol alters the neutrophil phenotype to overexpress integrin LFA-1, promoting ER^+^ cancer cell dissemination by activating cell-cell interaction (144). Meanwhile, it has been recently studied that neutrophils regulate diurnal transcription profiles in the lung, and promote the migration of B16F1 melanoma cells to the lungs (145). In CCL9-expressing CMT93 colon carcinoma, CCR1^+^ neutrophils secrete MMP9 to foster cancer foci, and in late phases of tumor the neutrophils recruit fibrocytes or induce differentiation from monocytes to fibrocytes which secrete MMP2, accommodating tumor cell colonization (86, 87). In short, the collaborative work of CCR1, MMP9, and MMP2 at metastatic sites promotes cancer metastasis (86, 87).

Neutrophil elastase traps (NETs) consist of extracellular decondensed DNA with granules and histones derived from neutrophils (146). Through a myriad of studies, it was explored that upon activation of neutrophils, neutrophil-derived NETs degrade virulence factors and trap bacteria within the vasculature, eventually killing them. Thus, NETs work as antimicrobial substances (146–148). It has also been elucidated that NETs play potent roles in tumor cell migration by trapping circulating cancer cells in vasculature and releasing secretory molecules by themselves (110). A study on the progression of H59 Lewis lung carcinoma after cecal ligation and puncture (CLP), represented as an alternative model of postsurgical infection, has proven that systemic sepsis induces neutrophil-derived NET formation in the hepatic sinusoid (110). Then the NETs enable stable retention of tumor cells and accelerate tumor growth within the liver (110). The link between trapped cancer cells by NETs and their proliferation in metastatic sites has been explicated in a metastatic MC38 tumor model followed by ischemia and reperfusion (I/R) injury, which is in an inevitable state after liver resection (82). Tumor hypoxia promotes NET formation within the metastatic site, and the NETs release the High mobility group box 1 (HMGB-1) protein (82). Secreted HMGB-1 activates TLR9, which encourages tumor progression via activation of related intracellular growth signaling pathways, involving phosphorylation of p38, Stat3, JNK and p65 of NF-kB (82). Moreover, it has been recently elucidated that NETs are involved in activation of dormant cancer cells in D2.0R mammary carcinoma (111). NET formation driven by LPS inflammation mediates laminin cleavage and thrombospondin-1 (Tsp-1) modulation by neutrophil elastase and NET-associated proteases (111, 149). This stimulates integrin α3β1 on dormant cancer cells and activates the FAK/ERK/MLCK/YAP signaling pathway to awaken cancer cells (111). Even in the absence of infection, 4T1 mammary cancer cells induce neutrophils to form NETs once they arrive at lung metastatic sites, promoting the expansion of disseminated cells (150). In ID8 ovarian cancer, the cancer cell-derived factors such as IL-8, GRO-a, GRO-b, and G-CSF enhance neutrophil influx to premetastatic omental niche and promote NET formation. In sequence, the NETs support tumor metastasis throughout trapping circulating ovarian cancer cells (151).

Meanwhile, the importance of considering cancer as a systemic disease has been highlighted again through its interaction with bones (152). In KP lung adenocarcinoma, the lung tumor activates Ocn+ osteoblasts via secretion of the soluble receptor for advanced glycation end products (sRAGE), which induces tumor infiltration of siglecF^high^ neutrophils and promotes tumor growth (152). These neutrophils represent a tumor-promoting transcriptional profile with upregulated expression of genes associated with angiogenesis (VEGFA, HIF1α, and SEMA4d), myeloid cell differentiation and recruitment (CSF1, CCL3, and MIF), extracellular matrix remodeling (ADAMDEC1, ADAM17, and many cathepsins), T cell suppression (PD-L1, FCGR2b, and HAVCR2), and tumor cell proliferation (TNF, TGFβ1, and IL-1α) (152). In contrast, the siglecF^high^ neutrophils downregulate genes involved in cytotoxicity (CD244, ITGAL, and Fas) (152). Furthermore, these neutrophils increase ROS production and foster monocyte differentiation into macrophages (152).

#### Anti-tumoral Functions of Neutrophils in Solid Tumors

The majority of the hitherto conducted researches indicate that neutrophils can only serve to promote tumor progression (Table 2; Figure 2). However, depending on the context, neutrophils suppress tumor metastasis by inhibiting malignant progression. In CT26 colon carcinoma, neutrophils inhibit the growth of G-CSF-producing cancer cells via contact-mediated cytostatic activity, but not G-CSF-nonproducing cancer cells (153). It has been recently revealed that the H_2_O_2_ secreted by neutrophils leads to tumor cell death, and TRPM2-mediated calcium influx acts as a go-between for this tumor killing process by neutrophils in AT3 and 4T1 breast cancer (114). In 4T1 mammary carcinoma and MMTV-PyMT spontaneous mammary carcinoma, entrained in the pre-metastatic lung prior to the arrival of metastatic cancer cells from primary sites, neutrophils play a cytotoxic function via physical contacts with cancer cells, secreting H_2_O_2_ and inhibiting the seeding of the cancer cells (115). Neutrophil-derived ROS secretion in B16F10 melanoma and Hepa1-6 hepatoma suppress IL-17^+^ γδ T cells which have pro-tumoral features, but not CD8^+^ T cells, in tumor niches (116). Neutrophils also have cytotoxic activity against CT26 colon cancer cells via production of granzyme B (113). Meanwhile, in B16F10 melanoma, T241 fibrosarcoma, LLC, and MMTV-PyMT-derived lung adenocarcinoma, tumor-induced TNF-α stimulates the NF-kB signaling pathway to express proto-oncogene MET in neutrophils (154). This enables the hepatocyte growth factor (HGF), also driven by the tumor, to bind to MET (154). HGF/MET signaling promotes neutrophil extravasation, induces iNOS and NO production, and thereby supports tumoricidal neutrophil function (154). In human PC3 prostate cancer and MDA-MB-231 breast cancer, bone marrow-derived CD11b^+^ Gr1^+^ cells which contain neutrophil populations mainly induce thrombospondin-1 (Tsp-1) in lung premetastatic sites, impairing tumor cell proliferation at the sites (117). It has been also reported that tumor-infiltrated neutrophils undergo functional changes and acquire an anti-tumoral phenotype, supporting T cell responses against tumor in early stages of human lung cancer (118). Photodynamic therapy (PDT) augments anti-tumor immunity and tumor regression by regulating the anti-tumoral functions of neutrophils (155).

Furthermore, neutrophils regulate pro-tumoral or anti-tumoral mechanisms depending on tumor stage. In LLC and AB12 mesothelioma, TANs from the early tumors are more cytotoxic toward tumor cells and produce higher levels of TNF-α, NO, and H_2_O_2_, while these expressions are downregulated in late stages of tumors in which TANs acquire an enhanced pro-tumoral phenotype (112). Although depletion of neutrophils in the early stages of tumor has no effect on tumor growth, depletion of neutrophils in late stages of tumor dramatically decreases tumor growth (112).

### Polarization of Tumor-Associated Neutrophils

According to a myriad of aforementioned studies on functions of neutrophils in diverse tumor circumstances, it has been well-established that TANs acquire pro-tumoral phenotype or anti-tumoral phenotype depending on related factors (147, 156). In AB12 mesothelioma and LKR lung carcinoma, TGF-β secreted by the tumor induces neutrophil polarization toward a pro-tumorigenic phenotype (156). Blockade of TGF-β attracts anti-tumorigenic neutrophils which release a large number of proinflammatory cytokines to infiltrate into the tumor microenvironment (156). Moreover, as the tumor develops, neutrophils display different functions regarding tumor growth through pro-tumoral or anti-tumoral mechanisms. IFN-β (type I IFN) differentiates neutrophils to achieve an anti-tumoral phenotype, reducing VEGF, and MMP9 expression (78, 79, 157). Inhibition of IFN-β endows TANs with pro-tumoral properties, and promotes growth and metastasis of B16F10 melanoma, MCA205 fibrosarcoma, 4T1 mammary carcinoma, CT26 colon carcinoma and Lewis lung carcinoma (78, 79, 157). *In vitro* study of BGC-823, MGC80-3, SGC-7901, and HGC-27 human gastric cancer cells has elucidated that interaction between HMGB1 secreted by the cancer cell-derived exosomes and toll-like receptor 4 (TLR4) on neutrophils fosters the formation of the autophagosome, inhibition of ROS production, and upregulation of MMP9 and VEGF in neutrophils, inducing polarization of neutrophils, promoting cancer cell migration (158).

## Clinical Aspects

### Prognostic Biomarkers

There have been a number of trials to predict cancer prognosis, including the TNM staging system established by The American Joint Committee on Cancer/Union Internationale Contre Ie Cancer (AJCC/UICC) (159). Through the TNM staging system, tumor prognostic information can be provided depending on tumor burden, the presence of cancer cells in lymph nodes (N) and event of distant metastases (M). Nonetheless, TNM provides limited capacity for accurate prediction (159). Cancer is a multidimensional disease, beyond difficulties in cure and prediction, which incurs many systemic alternations to be considered for effective treatment (159). One of the alternations emanates from the immune microenvironment. Reflecting the considerable impact of the immune system on tumor progression, the application of the immune parameter (Immunescore) has been introduced in disease classification to overcome the limitations of the traditional TNM staging system (159). As described above, presence of monocytes and neutrophils can be a double-edged sword, pro-tumoral or anti-tumoral, depending on the characteristics of tumors and applied therapies. In lung cancer, increased amount of monocytes within the tumor is associated with a poor survival rate, represented by progression-free survival (PFS) and overall survival (OS) of patients (36). In patients with colorectal cancer, profound influx of CCR2^+^ classical monocytes from the bone marrow to the circulatory system is correlated with worse clinical outcomes, showing accelerated liver metastasis (46). Reversely, in patients with melanoma, high frequency of classical monocytes allows us to predict favorable treatment response to anti-PD1 therapy and increased survival rates (160). Presence of TEMs and M2-polarized macrophages infiltrated in PDAC is associated with a high possibility of tumor recurrence and poor survival rates (161). In hepatitis B virus related hepatocellular carcinoma, high percentage of TEMs in peripheral blood monocytes represent poor overall survival and a shorter time to disease recurrence after resection (162). Changes in abundance between TEMs before and at 1 month after initial therapy also could serve as a biomarker in order to predict overall survival of hepatocellular carcinoma patients treated with sorafenib, a multi-kinase inhibitor of tumor angiogenesis (163). In breast cancer, endometrial cancer, prostate cancer, bladder cancer, ovary cancer, and urothelial cancer patients, high density of tumor-associated monocytes/macrophages (TAMs) has been reported to correlate with poor overall survival rates, while high density of TAMs in colorectal cancer patients shows longer overall survival (164). High density of TAMs is also associated with advanced tumor stages (III+IV) rather than with early stages (I+II) in breast cancer, oral cancer, and bladder cancer patients (164). However, there was no observed relation between TAMs and disease free survival rate in this clinical study (164).

Despite the controversial functions of neutrophils, neutrophil lymphocyte ratio (NLR) could be a potential biomarker for clinical use in some cases. After surgical removal of colorectal cancer (CRC), esophageal squamous cell carcinoma (ESCC), and PDAC, patients with lower values of NLR have a greater survival rate and reduced disease progression compared to patients with high NLR (165–167). When using everolimus for treatment of metastatic renal cell carcinoma (RCC), patients with low NLR also represent increased levels of both overall survival and PFS (168). Meanwhile, NLR inversely correlates with prostate-specific antigen (PSA) responsiveness to abiraterone acetate (abiraterone), a medication for metastatic castration-resistant prostate cancer patients (169, 170). In hepatocellular patients, tumor-infiltrated neutrophils represent upregulated PD-L1 expression (91). The ratio of PD-L1^+^ neutrophils to PD-1^+^ T cells helps better predict the disease-free survival of HCC patients (91). The NLR system is still under investigation across various cancer types, and it would be safe to be cautious to make an interpretation of disease prognosis with this system.

On one hand, counting TINs indicates controversial clinical outcomes. In RCC, presence of TINs has a negative impact on survival rates (171) and in melanoma patients, high amount of TINs mediated by activated pSTAT3 is linked to poor disease prognosis (172). Robust tumor infiltration of neutrophils also presents a negative disease progression of head and neck squamous cell carcinoma (HNSCC) (173). In the same manner, colorectal cancer patients with increased level of TINs are more likely to acquire a malignant phenotype of cancer and show adverse prognosis (174). Moreover, upon bevacizumab treatment (anti-VEGF therapy) for metastatic colorectal cancer patients, neutrophil infiltration engenders drastically low survival rates and represents a hostile clinical response against bevacizumab treatment (90). However, according to a couple of other clinical researches regarding influence of TINs on colorectal cancer prognosis, neutrophil infiltration to tumor tissue positively associates with favorable disease prognosis (175, 176) and with better responses to 5-FU-based chemotherapy (177). Interestingly, level of TINs may affect tumor prognosis differently depending on the sex of gastric cancer patients. Extensive amount of TINs reduces mortality risk of female patients while it does not affect male patients (178). Meanwhile, in non-small cell lung cancer (NSCLC) TINs do not represent any immediate impact on recurrence-free survival and overall survival (179).

Tests of functional single-nucleotide polymorphisms in genes regulating TAMs also enable us to predict clinical treatment outcomes (180). Through related trials, TBK1 rs7486100, CCL2 rs4586, CCL18 rs14304, and IRF3 rs2304205 have also been revealed to correlate with overall survival and progression free survival of metastatic colorectal cancer patients treated with bevacizumab (180).

### Therapeutic Applications

The CCL2-CCR2 chemokine axis plays a major role in recruitment of TAMs, which renders the immunosuppressive tumor microenvironment immunosuppressive and thereby promotes tumor progression (46). Conversely, inhibition of this axis restores anti-tumor immunity (46). Combination therapy of CCR2 inhibitor PF-04136309 with FOLFIRINOX chemotherapy for PDAC restores the anti-tumor immune microenvironment, preventing CCR2+ monocytes from emerging from the bone marrow (181). Carlumab is a human immunoglobulin G_1κ_ monoclonal antibody which specifically binds to human CCL2 with high affinity, leading to CCL2-CCR2 axis disruption (182–184). Clinical trials conducted for Carlumab in ovarian cancer, prostate cancer and other solid tumors with and without other chemotherapies such as docetaxel, gemcitabine, paclitaxel+carboplatin, or PLD has proven that Carlumab is well-tolerated but unfortunately fails to trigger significant tumor responses, since it could not sustain the long-term blockade of CCL2 (182–184). Since the CSF-1/CSF-1R axis is responsible for differentiation and survival of pro-tumoral TAMs, incessant efforts have been made to target CSF-1R to eliminate or repolarize TAMs (185). There are several CSF-1R inhibitors currently in clinical trials in many tumor types (186). Emactuzumab (RG7155) is a recombinant, humanized monoclonal antibody of IgG1 subclass, targeting CSF-1R expressed on macrophages (186). Clinical treatment of emactuzumab to patients with tenosynovial giant cell tumor shows durable tumor responses and functional improvement of patients with significant reduction of infiltrated macrophages in the tumor (186).

Another CSF-1R inhibitor is pexidartinib (PLX3397), a small-molecule inhibitor (187). As delineated above, preclinical studies in diverse solid tumors including mammary carcinoma, melanoma, lung carcinoma, pancreatic carcinoma, and glioma have proven that this molecule effectively blocks CSF-1R signaling, suppresses infiltration of macrophages into tumors, and accordingly restrains tumor progression (187–191). The dramatic tumor response to PLX3397 has provided a rationale to begin work on its clinical applications, currently ongoing in many solid tumors with and without combination with pembrolizumab, a monoclonal antibody targeting PD-1. According to a clinical case report, the progression of tenosynovial giant cell tumor was inhibited during non-surgical management with pexidartinib treatment (192).

In the context of tumor where neutrophils exert detrimental influence, the activation and homing of neutrophils need to be interrupted for better prognosis. Repertaxin is a small molecule inhibitor of CXCR1 and CXCR2 for blocking neutrophil trafficking (193). In patients with HER-2 negative metastatic breast cancer, treatment of repertaxin in combination with paclitaxel shows a durable tumor response with fine safety and tolerance. In this setting, an increased rate of neutropenia has not been observed, which needs to be evaluated further (194). Meanwhile, myeloid cell-derived IDO could be another attractive target for tumor regression since it shows suppressive activity on T cells (195). Preclinical research using MMTV-Neu breast tumor model has revealed that indoximod, a small molecule inhibitor of IDO, in combined use of paclitaxel, successfully induces tumor regression (195). As a clinical trial, targeting IDO with a peptide vaccine elicits long-lasting disease stabilization in lung cancer patients along with reduction of Treg frequency and increased cytotoxicity of CD8^+^ T cells to kill cancer cells (196). Clinical application of indoximod is also ongoing in metastatic solid tumor patients (197). However, it may be asked whether IDO is an effective target, since phase III ECHO301 trial of epacadostat, another inhibitor of IDO, with pembrolizumab for melanoma as a combination therapy failed, missing the first primary endpoint of improving PFS vs. pembrolizumab alone (198).

## Concluding Remarks

As thoroughly discussed in this review, tumor-associated monocytes and neutrophils are highly heterogeneous in a context dependent manner. Setting aside the need for the fine-tuning, we still have limited knowledge of their versatile functions in diverse tumor scenarios: cancer types, stages of disease, and applied therapies. In order to decipher these multifaceted roles of monocytes and neutrophils, there are several demands to be considered. First, we strongly suggest establishing orthotopic tumor models for preclinical studies. Ectopic tumor implantation has been conducted in many pieces of researches without consideration of organ settings. However, since the organ specific microenvironment, including different immune landscape, differently regulate tumor growth and progression, neglecting it undermines the validation of ectopic tumor models. Second, we also urge that researches be further progressed with development of applicable technologies such as single-cell RNA sequencing, intravital imaging, and mass cytometry. Application of advanced technological methods not only help in systemically understanding the heterogeneous and dynamic tumor microenvironment, but will also let us forecast disease prognosis and make therapeutic decisions with minimal side effects. Lastly, more clinical studies are required to validate prognostic markers and therapeutic agents.

With the fulfillment of these methodological and practical suggestions, we will be able to heighten our understanding of heterogeneous functions of monocytes and neutrophils in various tumor contexts, and further establish effective tumor therapies based on the comprehensive understanding.

## Author Contributions

KJ conceived the concept. JJ, YS, and KJ wrote the manuscript.

### Conflict of Interest Statement

The authors declare that the research was conducted in the absence of any commercial or financial relationships that could be construed as a potential conflict of interest.

## References

[1] Auffray C Sieweke MH Geissmann F. Blood monocytes: development, heterogeneity, and relationship with dendritic cells. Annu Rev Immunol. (2009) 27:669–92. 10.1146/annurev.immunol.021908.132557 19132917

[2] Dai MX Ryan GR Hapel AJ Dominguez MG Russell RG Kapp S. Targeted disruption of the mouse colony-stimulating factor 1 receptor gene results in osteopetrosis, mononuclear phagocyte deficiency, increased primitive progenitor cell frequencies, and reproductive defects. Blood. (2002) 99:111–20. 10.1182/blood.V99.1.111 11756160

[3] Guilliams M Mildner A Yona S. Developmental and functional heterogeneity of monocytes. Immunity. (2018) 49:595–613. 10.1016/j.immuni.2018.10.005 30332628

[4] Yona S Kim KW Wolf Y Mildner A Varol D Breker M. Fate mapping reveals origins and dynamics of monocytes and tissue macrophages under homeostasis. Immunity. (2013) 38:79–91. 10.1016/j.immuni.2012.12.001 23273845 PMC3908543

[5] Cros J Cagnard N Woollard K Patey N Zhang SY Senechal B. Human CD14dim monocytes patrol and sense nucleic acids and viruses via TLR7 and TLR8 receptors. Immunity. (2010) 33:375–86. 10.1016/j.immuni.2010.08.012 20832340 PMC3063338

[6] Geissmann F Jung S Littman DR. Blood monocytes consist of two principal subsets with distinct migratory properties. Immunity. (2003) 19:71–82. 10.1016/S1074-7613(03)00174-2 12871640

[7] Jung K Heishi T Khan OF Kowalski PS Incio J Rahbari NN. Ly6Clo monocytes drive immunosuppression and confer resistance to anti-VEGFR2 cancer therapy. J Clin Invest. (2017) 127:3039–51. 10.1172/JCI93182 28691930 PMC5531423

[8] Jung K Heishi T Incio J Huang Y Beech EY Pinter M Targeting CXCR4-dependent immunosuppressive Ly6C(low) monocytes improves antiangiogenic therapy in colorectal cancer. Proc Natl Acad Sci USA. (2017) 114:10455–60. 10.1073/pnas.1710754114 28900008 PMC5625928

[9] Auffray C Fogg D Garfa M Elain G Join-Lambert O Kayal S. Monitoring of blood vessels and tissues by a population of monocytes with patrolling behavior. Science. (2007) 317:666–70. 10.1126/science.1142883 17673663

[10] Murray PJ. Immune regulation by monocytes. Semin Immunol. (2018) 35:12–8. 10.1016/j.smim.2017.12.005 29290545

[11] Tamura A Hirai H Yokota A Kamio N Sato A Shoji T. C/EBPβ is required for survival of Ly6C(-) monocytes. Blood. (2017) 130:1809–18. 10.1182/blood-2017-03-772962 28807982 PMC5649551

[12] Thomas G Tacke R Hedrick CC Hanna RN. Nonclassical patrolling monocyte function in the vasculature. Arterioscler Thromb Vasc Biol. (2015) 35:1306–16. 10.1161/ATVBAHA.114.304650 25838429 PMC4441550

[13] Alder JK Georgantas RW Hildreth RL Kaplan IM Morisot S Yu X. Kruppel-like factor 4 is essential for inflammatory monocyte differentiation *in vivo*. J Immunol. (2008) 180:5645–52. 10.4049/jimmunol.180.8.5645 18390749 PMC3074963

[14] Kurotaki D Osato N Nishiyama A Yamamoto M Ban T Sato H. Essential role of the IRF8-KLF4 transcription factor cascade in murine monocyte differentiation. Blood. (2013) 121:1839–49. 10.1182/blood-2012-06-437863 23319570 PMC3591803

[15] Mildner A Schonheit J Giladi A David E Lara-Astiaso D Lorenzo-Vivas E. Genomic characterization of murine monocytes reveals C/EBPβ transcription factor dependence of Ly6C(-) cells. Immunity. (2017) 46:849–62.e7. 10.1016/j.immuni.2017.04.018 28514690

[16] Hanna RN Carlin LM Hubbeling HG Nackiewicz D Green AM Punt JA. The transcription factor NR4A1 (Nur77) controls bone marrow differentiation and the survival of Ly6C- monocytes. Nat Immunol. (2011) 12:778–85. 10.1038/ni.2063 21725321 PMC3324395

[17] Olingy CE Dinh HQ Hedrick CC. Monocyte heterogeneity and functions in cancer. J Leukoc Biol. (2019) 1–14. 10.1002/JLB.4RI0818-311R 30776148 PMC6658332

[18] Selimoglu-Buet D Riviere J Ghamlouch H Bencheikh L Lacout C Morabito M. A miR-150/TET3 pathway regulates the generation of mouse and human non-classical monocyte subset. Nat Commun. (2018) 9:5455. 10.1038/s41467-018-07801-x 30575719 PMC6303340

[19] Villani AC Satija R Reynolds G Sarkizova S Shekhar K Fletcher J. Single-cell RNA-seq reveals new types of human blood dendritic cells, monocytes, and progenitors. Science. (2017) 356:eaah4573. 10.1126/science.aah4573 28428369 PMC5775029

[20] Hamers AJ Dinh HQ Thomas GD Marcovecchio P Blatchley A Nakao CS. Human monocyte heterogeneity as revealed by high-dimensional mass cytometry. Arterioscler Thromb Vasc Biol. (2019) 39:25–36. 10.1161/ATVBAHA.118.311022 30580568 PMC6697379

[21] Jakubzick C Gautier EL Gibbings SL Sojka DK Schlitzer A Johnson TE. Minimal differentiation of classical monocytes as they survey steady-state tissues and transport antigen to lymph nodes. Immunity. (2013) 39:599–610. 10.1016/j.immuni.2013.08.007 24012416 PMC3820017

[22] Kim KW Williams JW Wang YT Ivanov S Gilfillan S Colonna M. MHC II+ resident peritoneal and pleural macrophages rely on IRF4 for development from circulating monocytes. J Exp Med. (2016) 213:1951–9. 10.1084/jem.20160486 27551152 PMC5030807

[23] Lavin Y Mortha A Rahman A Merad M. Regulation of macrophage development and function in peripheral tissues. Nat Rev Immunol. (2015) 15:731. 10.1038/nri3920 26603899 PMC4706379

[24] Ginhoux F Guilliams M. Tissue-resident macrophage ontogeny and homeostasis. Immunity. (2016) 44:439–49. 10.1016/j.immuni.2016.02.024 26982352

[25] Aras S Zaidi MR. TAMeless traitors: macrophages in cancer progression and metastasis. Br J Cancer. (2017) 117:1583–91. 10.1038/bjc.2017.356 29065107 PMC5729447

[26] Tran Janco JM Lamichhane P Karyampudi L Knutson KL. Tumor-infiltrating dendritic cells in cancer pathogenesis. J Immunol. (2015) 194:2985–91. 10.4049/jimmunol.1403134 25795789 PMC4369768

[27] Cortez-Retamozo V Etzrodt M Newton A Rauch PJ Chudnovskiy A Berger C. Origins of tumor-associated macrophages and neutrophils. Proc Natl Acad Sci USA. (2012) 109:2491–6. 10.1073/pnas.1113744109 22308361 PMC3289379

[28] Swirski FK Nahrendorf M Etzrodt M Wildgruber M Cortez-Retamozo V Panizzi P. Identification of splenic reservoir monocytes and their deployment to inflammatory sites. Science. (2009) 325:612–6. 10.1126/science.1175202 19644120 PMC2803111

[29] Cortez-Retamozo V Etzrodt M Newton A Ryan R Pucci F Sio SW. Angiotensin II drives the production of tumor-promoting macrophages. Immunity. (2013) 38:296–308. 10.1016/j.immuni.2012.10.015 23333075 PMC3582771

[30] Shand FH Ueha S Otsuji M Koid SS Shichino S Tsukui T. Tracking of intertissue migration reveals the origins of tumor-infiltrating monocytes. Proc Natl Acad Sci USA. (2014) 111:7771–6. 10.1073/pnas.1402914111 24825888 PMC4040600

[31] Salmon H Remark R Gnjatic S Merad M. Host tissue determinants of tumour immunity. Nat Rev Cancer. (2019) 19:215–27. 10.1038/s41568-019-0125-9 30867580 PMC7787168

[32] Qian BZ Li J Zhang H Kitamura T Zhang J Campion LR. CCL2 recruits inflammatory monocytes to facilitate breast-tumour metastasis. Nature. (2011) 475:222–5. 10.1038/nature10138 21654748 PMC3208506

[33] Long KB Gladney WL Tooker GM Graham K Fraietta JA Beatty GL. IFNγ and CCL2 cooperate to redirect tumor-infiltrating monocytes to degrade fibrosis and enhance chemotherapy efficacy in pancreatic carcinoma. Cancer Discov. (2016) 6:400–13. 10.1158/2159-8290.CD-15-1032 26896096 PMC4843521

[34] Keklikoglou I Cianciaruso C Guc E Squadrito ML Spring LM Tazzyman S. Chemotherapy elicits pro-metastatic extracellular vesicles in breast cancer models. Nat Cell Biol. (2019) 21:190–202. 10.1038/s41556-018-0256-3 30598531 PMC6525097

[35] Hanna RN Cekic C Sag D Tacke R Thomas GD Nowyhed H. Patrolling monocytes control tumor metastasis to the lung. Science. (2015) 350:985–90. 10.1126/science.aac9407 26494174 PMC4869713

[36] Lin GN Peng JW Xiao JJ Liu DY Xia ZJ. Prognostic impact of circulating monocytes and lymphocyte-to-monocyte ratio on previously untreated metastatic non-small cell lung cancer patients receiving platinum-based doublet. Med Oncol. (2014) 31:70. 10.1007/s12032-014-0070-0 24927957

[37] Feng F Zheng G Wang Q Liu S Liu Z Xu G. Low lymphocyte count and high monocyte count predicts poor prognosis of gastric cancer. BMC Gastroenterol. (2018) 18:148. 10.1186/s12876-018-0877-9 30305076 PMC6180580

[38] Chittezhath M Dhillon MK Lim JY Laoui D Shalova IN Teo YL. Molecular profiling reveals a tumor-promoting phenotype of monocytes and macrophages in human cancer progression. Immunity. (2014) 41:815–29. 10.1016/j.immuni.2014.09.014 25453823

[39] Porrello A Leslie PL Harrison EB Gorentla BK Kattula S Ghosh SK. Factor XIIIA-expressing inflammatory monocytes promote lung squamous cancer through fibrin cross-linking. Nat Commun. (2018) 9:1988. 10.1038/s41467-018-04355-w 29777108 PMC5959879

[40] Chen Y Huang Y Reiberger T Duyverman AM Huang P Samuel R. Differential effects of sorafenib on liver versus tumor fibrosis mediated by stromal-derived factor 1 alpha/C-X-C receptor type 4 axis and myeloid differentiation antigen-positive myeloid cell infiltration in mice. Hepatology. (2014) 59:1435–47. 10.1002/hep.26790 24242874 PMC3966948

[41] Sidibe A Ropraz P Jemelin S Emre Y Poittevin M Pocard M. Angiogenic factor-driven inflammation promotes extravasation of human proangiogenic monocytes to tumours. Nat Commun. (2018) 9:355. 10.1038/s41467-017-02610-0 29367702 PMC5783934

[42] Plebanek MP Angeloni NL Vinokour E Li J Henkin A Martinez-Marin D. Pre-metastatic cancer exosomes induce immune surveillance by patrolling monocytes at the metastatic niche. Nat Commun. (2017) 8:1319. 10.1038/s41467-017-01433-3 29105655 PMC5673063

[43] Lavin Y Kobayashi S Leader A Amir ED Elefant N Bigenwald C. Innate immune landscape in early lung adenocarcinoma by paired single-cell analyses. Cell. (2017) 169:750–65.e17. 10.1016/j.cell.2017.04.014 28475900 PMC5737939

[44] Kubo H Mensurado S Gonçalves-Sousa N Serre K Silva-Santos B. Primary tumors limit metastasis formation through induction of IL15-mediated cross-talk between patrolling monocytes and NK cells. Cancer Immunol Res. (2017) 5:812–20. 10.1158/2326-6066.CIR-17-0082 28811289

[45] Sanford DE Belt BA Panni RZ Mayer A Deshpande AD Carpenter D. Inflammatory monocyte mobilization decreases patient survival in pancreatic cancer: a role for targeting the CCL2/CCR2 axis. Clin Cancer Res. (2013) 19:3404–15. 10.1158/1078-0432.CCR-13-0525 23653148 PMC3700620

[46] Grossman JG Nywening TM Belt BA Panni RZ Krasnick BA DeNardo DG. Recruitment of CCR2(+) tumor associated macrophage to sites of liver metastasis confers a poor prognosis in human colorectal cancer. Oncoimmunology. (2018) 7:e1470729. 10.1080/2162402X.2018.1470729 30228938 PMC6140580

[47] Harney AS Arwert EN Entenberg D Wang Y Guo P Qian BZ. Real-time imaging reveals local, transient vascular permeability, and tumor cell intravasation stimulated by TIE2hi macrophage-derived VEGFA. Cancer Discov. (2015) 5:932–43. 10.1158/2159-8290.CD-15-0012 26269515 PMC4560669

[48] Nakasone ES Askautrud HA Kees T Park JH Plaks V Ewald AJ. Imaging tumor-stroma interactions during chemotherapy reveals contributions of the microenvironment to resistance. Cancer Cell. (2012) 21:488–503. 10.1016/j.ccr.2012.02.017 22516258 PMC3332002

[49] Kalbasi A Komar C Tooker GM Liu M Lee JW Gladney WL. Tumor-derived CCL2 mediates resistance to radiotherapy in pancreatic ductal adenocarcinoma. Clin Cancer Res. (2017) 23:137–48. 10.1158/1078-0432.CCR-16-0870 27354473 PMC5195913

[50] Bonapace L Coissieux MM Wyckoff J Mertz KD Varga Z Junt T. Cessation of CCL2 inhibition accelerates breast cancer metastasis by promoting angiogenesis. Nature. (2014) 515:130–3. 10.1038/nature13862 25337873

[51] Nagathihalli NS Castellanos JA Shi C Beesetty Y Reyzer ML Caprioli R. Signal transducer and activator of transcription 3, mediated remodeling of the tumor microenvironment results in enhanced tumor drug delivery in a mouse model of pancreatic cancer. Gastroenterology. (2015) 149:1932–43.e9. 10.1053/j.gastro.2015.07.058 26255562 PMC4863449

[52] Long KB Tooker G Tooker E Luque SL Lee JW Pan X. IL6 receptor blockade enhances chemotherapy efficacy in pancreatic ductal adenocarcinoma. Mol Cancer Ther. (2017) 16:1898–908. 10.1158/1535-7163.MCT-16-0899 28611107 PMC5587413

[53] Corcoran RB Contino G Deshpande V Tzatsos A Conrad C Benes CH. Plays a critical role in -induced pancreatic tumorigenesis. Cancer Res. (2011) 71:5020–9. 10.1158/0008-5472.CAN-11-0908 21586612 PMC3693754

[54] Incio J Ligibel JA McManus DT Suboj P Jung K Kawaguchi K. Obesity promotes resistance to anti-VEGF therapy in breast cancer by up-regulating IL-6 and potentially FGF-2. Sci Transl Med. (2018) 10:eaag0945. 10.1126/scitranslmed.aag0945 29540614 PMC5936748

[55] Jiang H Hegde S DeNardo DG. Tumor-associated fibrosis as a regulator of tumor immunity and response to immunotherapy. Cancer Immunol Immunother. (2017) 66:1037–48. 10.1007/s00262-017-2003-1 28451791 PMC5603233

[56] Cox TR Erler JT. Molecular pathways: connecting fibrosis and solid tumor metastasis. Clin Cancer Res. (2014) 20:3637–43. 10.1158/1078-0432.CCR-13-1059 25028505

[57] Rahbari NN Kedrin D Incio J Liu H Ho WW Nia HT. Anti-VEGF therapy induces ECM remodeling and mechanical barriers to therapy in colorectal cancer liver metastases. Sci Transl Med. (2016) 8:360ra135. 10.1126/scitranslmed.aaf5219 27733559 PMC5457741

[58] Incio J Liu H Suboj P Chin SM Chen IX Pinter M. Obesity-induced inflammation and desmoplasia promote pancreatic cancer progression and resistance to chemotherapy. Cancer Discov. (2016) 6:852–69. 10.1158/2159-8290.CD-15-1177 27246539 PMC4972679

[59] Satoh T Nakagawa K Sugihara F Kuwahara R Ashihara M Yamane F. Identification of an atypical monocyte and committed progenitor involved in fibrosis. Nature. (2017) 541:96–101. 10.1038/nature20611 28002407

[60] Carlin LM Stamatiades EG Auffray C Hanna RN Glover L Vizcay-Barrena G. Nr4a1-dependent Ly6C(low) monocytes monitor endothelial cells and orchestrate their disposal. Cell. (2013) 153:362–75. 10.1016/j.cell.2013.03.010 23582326 PMC3898614

[61] Chen IX Chauhan VP Posada J Ng MR Wu MW Adstamongkonkul P Blocking CXCR4 alleviates desmoplasia, increases T-lymphocyte infiltration, and improves immunotherapy in metastatic breast cancer. Proc Natl Acad Sci USA. (2019) 116:4558–4566. 10.1158/1538-7445.SABCS18-2744 PMC6410779 30700545

[62] Romano E Kusio-Kobialka M Foukas PG Baumgaertner P Meyer C Ballabeni P. Ipilimumab-dependent cell-mediated cytotoxicity of regulatory T cells *ex vivo* by nonclassical monocytes in melanoma patients. Proc Natl Acad Sci USA. (2015) 112:6140–5. 10.1073/pnas.1417320112 25918390 PMC4434760

[63] De Palma M Murdoch C Venneri MA Naldini L Lewis CE. Tie2-expressing monocytes: regulation of tumor angiogenesis and therapeutic implications. Trends Immunol. (2007) 28:519–24. 10.1016/j.it.2007.09.004 17981504

[64] De Palma M Venneri MA Roca C Naldini L. Targeting exogenous genes to tumor angiogenesis by transplantation of genetically modified hematopoietic stem cells. Nat Med. (2003) 9:789. 10.1038/nm871 12740570

[65] De Palma M Venneri MA Galli R Sergi LS Politi LS Sampaolesi M. Tie2 identifies a hematopoietic lineage of proangiogenic monocytes required for tumor vessel formation and a mesenchymal population of pericyte progenitors. Cancer Cell. (2005) 8:211–26. 10.1016/j.ccr.2005.08.002 16169466

[66] Venneri MA Palma MD Ponzoni M Pucci F Scielzo C Zonari E. Identification of proangiogenic TIE2-expressing monocytes (TEMs) in human peripheral blood and cancer. Blood. (2007) 109:5276–85. 10.1182/blood-2006-10-053504 17327411

[67] Murdoch C Tazzyman S Webster S Lewis CE. Expression of Tie-2 by human monocytes and their responses to angiopoietin-2. J Immunol. (2007) 178:7405–11. 10.4049/jimmunol.178.11.7405 17513791

[68] Coffelt SB Tal AO Scholz A De Palma M Patel S Urbich C. Angiopoietin-2 regulates gene expression in TIE2-expressing monocytes and augments their inherent proangiogenic functions. Cancer Res. (2010) 70:5270–80. 10.1158/0008-5472.CAN-10-0012 20530679

[69] Lee J Song J Kwon ES Jo S Kang MK Kim YJ. CTHRC1 promotes angiogenesis by recruiting Tie2-expressing monocytes to pancreatic tumors. Exp Molecul Med. (2016) 48:e261. 10.1038/emm.2016.87 27686285 PMC5050301

[70] Lewis CE De Palma M Naldini L. Tie2-expressing monocytes and tumor angiogenesis: regulation by hypoxia and angiopoietin-2. Cancer Res. (2007) 67:8429–32. 10.1158/0008-5472.CAN-07-1684 17875679

[71] Mazzieri R Pucci F Moi D Zonari E Ranghetti A Berti A. Targeting the ANG2/TIE2 axis inhibits tumor growth and metastasis by impairing angiogenesis and disabling rebounds of proangiogenic myeloid cells. Cancer Cell. (2011) 19:512–26. 10.1016/j.ccr.2011.02.005 21481792

[72] Nicolas-Avila JA Adrover JM Hidalgo A. Neutrophils in homeostasis, immunity, and cancer. Immunity. (2017) 46:15–28. 10.1016/j.immuni.2016.12.012 28099862

[73] Coffelt SB Wellenstein MD de Visser KE. Neutrophils in cancer: neutral no more. Nat Rev Cancer. (2016) 16:431–46. 10.1038/nrc.2016.52 27282249

[74] Kolaczkowska E Kubes P. Neutrophil recruitment and function in health and inflammation. Nat Rev Immunol. (2013) 13:159–75. 10.1038/nri3399 23435331

[75] Fiedler K Brunner C Mechanisms controlling hematopoiesis. In: Lawrie CH editor. Hematology-Science and Practice. London: IntechOpen (2012). 10.5772/33749

[76] Evrard M Kwok IWH Chong SZ Teng KWW Becht E Chen J. Developmental analysis of bone marrow neutrophils reveals populations specialized in expansion, trafficking, and effector functions. Immunity. (2018) 48:364–379.e8. 10.1016/j.immuni.2018.02.002 29466759

[77] Phan VT Wu X Cheng JH Sheng RX Chung AS Zhuang G. Oncogenic RAS pathway activation promotes resistance to anti-VEGF therapy through G-CSF-induced neutrophil recruitment. Proc Natl Acad Sci USA. (2013) 110:6079–84. 10.1073/pnas.1303302110 23530240 PMC3625253

[78] Jablonska J Leschner S Westphal K Lienenklaus S Weiss S. Neutrophils responsive to endogenous IFN-beta regulate tumor angiogenesis and growth in a mouse tumor model. J Clin Invest. (2010) 120:1151–64. 10.1172/JCI37223 20237412 PMC2846036

[79] Wu CF Andzinski L Kasnitz N Kroger A Klawonn F Lienenklaus S. The lack of type I interferon induces neutrophil-mediated pre-metastatic niche formation in the mouse lung. Int J Cancer. (2015) 137:837–47. 10.1002/ijc.29444 25604426

[80] Gong F Peng X Luo C Shen G Zhao C Zou L. Cathepsin B as a potential prognostic and therapeutic marker for human lung squamous cell carcinoma. Molecul Cancer. (2013) 12:125. 10.1186/1476-4598-12-125 24139065 PMC3834881

[81] Mangerich A Knutson CG Parry NM Muthupalani S Ye W Prestwich E. Infection-induced colitis in mice causes dynamic and tissue-specific changes in stress response and DNA damage leading to colon cancer. Proc Natl Acad Sci USA. (2012) 109:E1820–9. 10.1073/pnas.1207829109 22689960 PMC3390855

[82] Tohme S Yazdani HO Al-Khafaji AB Chidi AP Loughran P Mowen K. Neutrophil extracellular traps promote the development and progression of liver metastases after surgical stress. Cancer Res. (2016) 76:1367–80. 10.1158/0008-5472.CAN-15-1591 26759232 PMC4794393

[83] Houghton AM Rzymkiewicz DM Ji H Gregory AD Egea EE Metz HE. Neutrophil elastase-mediated degradation of IRS-1 accelerates lung tumor growth. Nat Med. (2010) 16:219–23. 10.1038/nm.2084 20081861 PMC2821801

[84] Szczerba BM Castro-Giner F Vetter M Krol I Gkountela S Landin J. Neutrophils escort circulating tumour cells to enable cell cycle progression. Nature. (2019) 566:553–57. 10.1038/s41586-019-0915-y 30728496

[85] Liang W Li Q Ferrara N. Metastatic growth instructed by neutrophil-derived transferrin. Proc Natl Acad Sci USA. (2018) 115:11060–5. 10.1073/pnas.1811717115 30301793 PMC6205468

[86] Hirai H Fujishita T Kurimoto K Miyachi H Kitano S Inamoto S. CCR1-mediated accumulation of myeloid cells in the liver microenvironment promoting mouse colon cancer metastasis. Clin Exp Metastasis. (2014) 31:977–89. 10.1007/s10585-014-9684-z 25326065 PMC4256518

[87] Kitamura T Fujishita T Loetscher P Revesz L Hashida H Kizaka-Kondoh S. Inactivation of chemokine (C-C motif) receptor 1 (CCR1) suppresses colon cancer liver metastasis by blocking accumulation of immature myeloid cells in a mouse model. Proc Natl Acad Sci USA. (2010) 107:13063–8. 10.1073/pnas.1002372107 20616008 PMC2919974

[88] Chen Y Ramjiawan RR Reiberger T Ng MR Hato T Huang Y. CXCR4 inhibition in tumor microenvironment facilitates anti-programmed death receptor-1 immunotherapy in sorafenib-treated hepatocellular carcinoma in mice. Hepatology. (2015) 61:1591–602. 10.1002/hep.27665 25529917 PMC4406806

[89] Hiratsuka S Duda DG Huang Y Goel S Sugiyama T Nagasawa T. C-X-C receptor type 4 promotes metastasis by activating p38 mitogen-activated protein kinase in myeloid differentiation antigen (Gr-1)-positive cells. Proc Natl Acad Sci USA. (2011) 108:302–7. 10.1073/pnas.1016917108 21173223 PMC3017172

[90] Schiffmann LM Fritsch M Gebauer F Gunther SD Stair NR Seeger JM. Tumour-infiltrating neutrophils counteract anti-VEGF therapy in metastatic colorectal cancer. Br J Cancer. (2019) 120:69–78. 10.1038/s41416-018-0198-3 30377339 PMC6325148

[91] He G Zhang H Zhou J Wang B Chen Y Kong Y. Peritumoural neutrophils negatively regulate adaptive immunity via the PD-L1/PD-1 signalling pathway in hepatocellular carcinoma. J Exp Clin Cancer Res. (2015) 34:141. 10.1186/s13046-015-0256-0 26581194 PMC4652417

[92] Koyama S Akbay EA Li YY Aref AR Skoulidis F Herter-Sprie GS. STK11/LKB1 deficiency promotes neutrophil recruitment and proinflammatory cytokine production to suppress T-cell activity in the lung tumor microenvironment. Cancer Res. (2016) 76:999–1008. 10.1158/0008-5472.CAN-15-1439 26833127 PMC4775354

[93] Coffelt SB Kersten K Doornebal CW Weiden J Vrijland K Hau CS. IL-17-producing γ*δ* T cells and neutrophils conspire to promote breast cancer metastasis. Nature. (2015) 522:345–8. 10.1038/nature14282 25822788 PMC4475637

[94] Michaeli J Shaul ME Mishalian I Hovav AH Levy L Zolotriov L. Tumor-associated neutrophils induce apoptosis of non-activated CD8 T-cells in a TNFα and NO-dependent mechanism, promoting a tumor-supportive environment. Oncoimmunology. (2017) 6:e1356965. 10.1080/2162402X.2017.1356965 29147615 PMC5674962

[95] Rotondo R Barisione G Mastracci L Grossi F Orengo AM Costa R. IL-8 induces exocytosis of arginase 1 by neutrophil polymorphonuclears in nonsmall cell lung cancer. Int J Cancer. (2009) 125:887–93. 10.1002/ijc.24448 19431148

[96] Mishalian I Bayuh R Eruslanov E Michaeli J Levy L Zolotarov L. Neutrophils recruit regulatory T-cells into tumors via secretion of CCL17–a new mechanism of impaired antitumor immunity. Int J Cancer. (2014) 135:1178–86. 10.1002/ijc.28770 24501019

[97] Shojaei F Singh M Thompson JD Ferrara N. Role of Bv8 in neutrophil-dependent angiogenesis in a transgenic model of cancer progression. Proc Natl Acad Sci USA. (2008) 105:2640–5. 10.1073/pnas.0712185105 18268320 PMC2268189

[98] Qu X Zhuang G Yu L Meng G Ferrara N. Induction of Bv8 expression by granulocyte colony-stimulating factor in CD11b+Gr1+ cells: key role of Stat3 signaling. J Biol Chem. (2012) 287:19574–84. 10.1074/jbc.M111.326801 22528488 PMC3365993

[99] Nozawa H Chiu C Hanahan D. Infiltrating neutrophils mediate the initial angiogenic switch in a mouse model of multistage carcinogenesis. Proc Natl Acad Sci USA. (2006) 103:12493–8. 10.1073/pnas.0601807103 16891410 PMC1531646

[100] Bergers G Brekken R McMahon G Vu TH Itoh T Tamaki K. Matrix metalloproteinase-9 triggers the angiogenic switch during carcinogenesis. Nat Cell Biol. (2000) 2:737–44. 10.1038/35036374 11025665 PMC2852586

[101] Deryugina EI Zajac E Juncker-Jensen A Kupriyanova TA Welter L Quigley JP. Tissue-infiltrating neutrophils constitute the major in vivo source of angiogenesis-inducing MMP-9 in the tumor microenvironment. Neoplasia. (2014) 16:771–88. 10.1016/j.neo.2014.08.013 25379015 PMC4212255

[102] Bekes EM Schweighofer B Kupriyanova TA Zajac E Ardi VC Quigley JP. Tumor-recruited neutrophils and neutrophil TIMP-free MMP-9 regulate coordinately the levels of tumor angiogenesis and efficiency of malignant cell intravasation. Am J Pathol. (2011) 179:1455–70. 10.1016/j.ajpath.2011.05.031 21741942 PMC3157227

[103] Jablonska E Piotrowski L Jablonski J Grabowska Z. VEGF in the culture of PMN and the serum in oral cavity cancer patients. Oral Oncology. (2002) 38:605–9. 10.1016/S1368-8375(01)00110-5 12167439

[104] Gordon-Weeks AN Lim SY Yuzhalin AE Jones K Markelc B Kim KJ. Neutrophils promote hepatic metastasis growth through fibroblast growth factor 2-dependent angiogenesis in mice. Hepatology. (2017) 65:1920–35. 10.1002/hep.29088 28133764

[105] Spiegel A Brooks MW Houshyar S Reinhardt F Ardolino M Fessler E. Neutrophils suppress intraluminal NK cell-mediated tumor cell clearance and enhance extravasation of disseminated carcinoma cells. Cancer Discov. (2016) 6:630–49. 10.1158/2159-8290.CD-15-1157 27072748 PMC4918202

[106] Wculek SK Malanchi I. Neutrophils support lung colonization of metastasis-initiating breast cancer cells. Nature. (2015) 528:413–7. 10.1038/nature16140 26649828 PMC4700594

[107] Huh SJ Liang S Sharma A Dong C Robertson GP. Transiently entrapped circulating tumor cells interact with neutrophils to facilitate lung metastasis development. Cancer Res. (2010) 70:6071–82. 10.1158/0008-5472.CAN-09-4442 20610626 PMC2905495

[108] Li S Cong X Gao H Lan X Li Z Wang W Tumor-associated neutrophils induce EMT by IL-17a to promote migration and invasion in gastric cancer cells. J Exp Clin Cancer Res. (2019) 38:177 10.1186/s13046-019-1168-1 30616627 PMC6323742

[109] Spicer JD McDonald B Cools-Lartigue JJ Chow SC Giannias B Kubes P. Neutrophils promote liver metastasis via Mac-1-mediated interactions with circulating tumor cells. Cancer Res. (2012) 72:3919–27. 10.1158/0008-5472.CAN-11-2393 22751466

[110] Cools-Lartigue J Spicer J McDonald B Gowing S Chow S Giannias B Neutrophil extracellular traps sequester circulating tumor cells and promote metastasis. J Clin Invest. (2013) 123:3446–3458. 10.1158/1538-7445.AM2012-2972 PMC3726160 23863628

[111] Albrengues J Shields MA Ng D Park CG Ambrico A Poindexter ME. Neutrophil extracellular traps produced during inflammation awaken dormant cancer cells in mice. Science. (2018) 361:eaao4227. 10.1126/science.aao4227 30262472 PMC6777850

[112] Mishalian I Bayuh R Levy L Zolotarov L Michaeli J Fridlender ZG. Tumor-associated neutrophils (TAN) develop pro-tumorigenic properties during tumor progression. Cancer Immunol Immunother. (2013) 62:1745–56. 10.1007/s00262-013-1476-9 24092389 PMC11028422

[113] Martin A Seignez C Racoeur C Isambert N Mabrouk N Scagliarini A. Tumor-derived granzyme B-expressing neutrophils acquire antitumor potential after lipid A treatment. Oncotarget. (2018) 9:28364. 10.18632/oncotarget.25342 29983866 PMC6033356

[114] Gershkovitz M Caspi Y Fainsod-Levi T Katz B Michaeli J Khawaled S. TRPM2 mediates neutrophil killing of disseminated tumor cells. Cancer Res. (2018) 78:2680–90. 10.1158/0008-5472.CAN-17-3614 29490946

[115] Granot Z Henke E Comen EA King TA Norton L Benezra R. Tumor entrained neutrophils inhibit seeding in the premetastatic lung. Cancer Cell. (2011) 20:300–14. 10.1016/j.ccr.2011.08.012 21907922 PMC3172582

[116] Mensurado S Rei M Lanca T Ioannou M Goncalves-Sousa N Kubo H. Tumor-associated neutrophils suppress pro-tumoral IL-17+ γ*δ* T cells through induction of oxidative stress. PLoS Biol. (2018) 16:e2004990. 10.1371/journal.pbio.2004990 29750788 PMC5965901

[117] Catena R Bhattacharya N El Rayes T Wang S Choi H Gao D. Bone marrow-derived Gr1+ cells can generate a metastasis-resistant microenvironment via induced secretion of thrombospondin-1. Cancer Discov. (2013) 3:578–89. 10.1158/2159-8290.CD-12-0476 23633432 PMC3672408

[118] Eruslanov EB Bhojnagarwala PS Quatromoni JG Stephen TL Ranganathan A Deshpande C. Tumor-associated neutrophils stimulate T cell responses in early-stage human lung cancer. J Clin Invest. (2014) 124:5466–80. 10.1172/JCI77053 25384214 PMC4348966

[119] Steele CW Karim SA Leach JDG Bailey P Upstill-Goddard R Rishi L. CXCR2 inhibition profoundly suppresses metastases and augments immunotherapy in pancreatic ductal adenocarcinoma. Cancer Cell. (2016) 29:832–45. 10.1016/j.ccell.2016.04.014 27265504 PMC4912354

[120] Jamieson T Clarke M Steele CW Samuel MS Neumann J Jung A. Inhibition of CXCR2 profoundly suppresses inflammation-driven and spontaneous tumorigenesis. J Clin Invest. (2012) 122:3127–44. 10.1172/JCI61067 22922255 PMC3428079

[121] Beauvillain C Cunin P Doni A Scotet M Jaillon S Loiry ML. CCR7 is involved in the migration of neutrophils to lymph nodes. Blood. (2011) 117:1196–204. 10.1182/blood-2009-11-254490 21051556

[122] Benevides L da Fonseca DM Donate PB Tiezzi DG De Carvalho DD de Andrade JM. IL17 promotes mammary tumor progression by changing the behavior of tumor cells and eliciting tumorigenic neutrophils recruitment. Cancer Res. (2015) 75:3788–99. 10.1158/0008-5472.CAN-15-0054 26208902 PMC8101363

[123] Akbay EA Koyama S Liu Y Dries R Bufe LE Silkes M. Interleukin-17A promotes lung tumor progression through neutrophil attraction to tumor sites and mediating resistance to PD-1 blockade. J Thorac Oncol. (2017) 12:1268–79. 10.1016/j.jtho.2017.04.017 28483607 PMC5532066

[124] Charles KA Kulbe H Soper R Escorcio-Correia M Lawrence T Schultheis A. The tumor-promoting actions of TNF-alpha involve TNFR1 and IL-17 in ovarian cancer in mice and humans. J Clin Invest. (2009) 119:3011–23. 10.1172/JCI39065 19741298 PMC2752076

[125] Powell D Lou M Barros Becker F Huttenlocher A. Cxcr1 mediates recruitment of neutrophils and supports proliferation of tumor-initiating astrocytes *in vivo*. Sci Rep. (2018) 8:13285. 10.1038/s41598-018-31675-0 30185911 PMC6125480

[126] Czepielewski RS Porto BN Rizzo LB Roesler R Abujamra AL Pinto LG. Gastrin-releasing peptide receptor (GRPR) mediates chemotaxis in neutrophils. Proc Natl Acad Sci USA. (2012) 109:547–52. 10.1073/pnas.1110996109 22203955 PMC3258617

[127] Cornelio D Roesler R Schwartsmann G. Gastrin-releasing peptide receptor as a molecular target in experimental anticancer therapy. Ann Oncol. (2007) 18:1457–66. 10.1093/annonc/mdm058 17351255

[128] Soler-Cardona A Forsthuber A Lipp K Ebersberger S Heinz M Schossleitner K. CXCL5 facilitates melanoma cell-neutrophil interaction and lymph node metastasis. J Invest Dermatol. (2018) 138:1627–35. 10.1016/j.jid.2018.01.035 29474942

[129] Chen Y Liu YC Sung YC Ramjiawan RR Lin TT Chang CC. Overcoming sorafenib evasion in hepatocellular carcinoma using CXCR4-targeted nanoparticles to co-deliver MEK-inhibitors. Sci Rep. (2017) 7:44123. 10.1038/srep44123 28276530 PMC5343435

[130] Sung YC Liu YC Chao PH Chang CC Jin PR Lin TT. Combined delivery of sorafenib and a MEK inhibitor using CXCR4-targeted nanoparticles reduces hepatic fibrosis and prevents tumor development. Theranostics. (2018) 8:894–905. 10.7150/thno.21168 29463989 PMC5817100

[131] Yang J Kumar A Vilgelm AE Chen SC Ayers GD Novitskiy SV. Loss of CXCR4 in Myeloid cells enhances antitumor immunity and reduces melanoma growth through NK cell and FASL mechanisms. Cancer Immunol Res. (2018) 6:1186–98. 10.1158/2326-6066.CIR-18-0045 30108045 PMC6170679

[132] Rice CM Davies LC Subleski JJ Maio N Gonzalez-Cotto M Andrews C. Tumour-elicited neutrophils engage mitochondrial metabolism to circumvent nutrient limitations and maintain immune suppression. Nat Commun. (2018) 9:5099. 10.1038/s41467-018-07505-2 30504842 PMC6269473

[133] Zhu YP Padgett L Dinh HQ Marcovecchio P Blatchley A Wu R. Identification of an early unipotent neutrophil progenitor with pro-tumoral activity in mouse and human bone marrow. Cell Rep. (2018) 24:2329–41.e8. 10.1016/j.celrep.2018.07.097 30157427 PMC6542273

[134] Bronte V Brandau S Chen SH Colombo MP Frey AB Greten TF. Recommendations for myeloid-derived suppressor cell nomenclature and characterization standards. Nat Commun. (2016) 7:12150. 10.1038/ncomms12150 27381735 PMC4935811

[135] Mazzone M Bergers G. Regulation of blood and lymphatic vessels by immune cells in tumors and metastasis. Annu Rev Physiol. (2019) 81:535–60. 10.1146/annurev-physiol-020518-114721 30742781 PMC6589442

[136] Faget J Groeneveld S Boivin G Sankar M Zangger N Garcia M. Neutrophils and snail orchestrate the establishment of a pro-tumor microenvironment in lung cancer. Cell Rep. (2017) 21:3190–204. 10.1016/j.celrep.2017.11.052 29241546

[137] Hsu DS Wang HJ Tai SK Chou CH Hsieh CH Chiu PH. Acetylation of snail modulates the cytokinome of cancer cells to enhance the recruitment of macrophages. Cancer Cell. (2014) 26:534–48. 10.1016/j.ccell.2014.09.002 25314079

[138] Kudo-Saito C Shirako H Takeuchi T Kawakami Y. Cancer metastasis is accelerated through immunosuppression during Snail-induced EMT of cancer cells. Cancer Cell. (2009) 15:195–206. 10.1016/j.ccr.2009.01.023 19249678

[139] Tazawa H Okada F Kobayashi T Tada M Mori Y Une Y. Infiltration of neutrophils is required for acquisition of metastatic phenotype of benign murine fibrosarcoma cells: implication of inflammation-associated carcinogenesis and tumor progression. Am J Pathol. (2003) 163:2221–32. 10.1016/S0002-9440(10)63580-8 14633597 PMC1892401

[140] Fan Z McArdle S Marki A Mikulski Z Gutierrez E Engelhardt B. Neutrophil recruitment limited by high-affinity bent β2 integrin binding ligand in cis. Nat Commun. (2016) 7:12658. 10.1038/ncomms12658 27578049 PMC5013657

[141] Ley K Hoffman HM Kubes P Cassatella MA Zychlinsky A Hedrick CC. Neutrophils: new insights and open questions. Sci Immunol. (2018) 3:eaat4579. 10.1126/sciimmunol.aat4579 30530726

[142] Hanahan D Weinberg RA. Hallmarks of cancer: the next generation. Cell. (2011) 144:646–74. 10.1016/j.cell.2011.02.013 21376230

[143] Chen MB Hajal C Benjamin DC Yu C Azizgolshani H Hynes RO Kamm RD. Inflamed neutrophils sequestered at entrapped tumor cells via chemotactic confinement promote tumor cell extravasation. Proc Natl Acad Sci USA. (2018) 115:7022–7. 10.1073/pnas.1715932115 29915060 PMC6142213

[144] Vazquez Rodriguez G Abrahamsson A Jensen LD Dabrosin C. Estradiol promotes breast cancer cell migration via recruitment and activation of neutrophils. Cancer Immunol Res. (2017) 5:234–47. 10.1158/2326-6066.CIR-16-0150 28159748

[145] Casanova-Acebes M Nicolas-Avila JA Li JL Garcia-Silva S Balachander A Rubio-Ponce A. Neutrophils instruct homeostatic and pathological states in naive tissues. J Exp Med. (2018) 215:2778–95. 10.1084/jem.20181468 30282719 PMC6219739

[146] Brinkmann V Reichard U Goosmann C Fauler B Uhlemann Y Weiss DS. Neutrophil extracellular traps kill bacteria. Science. (2004) 303:1532–5. 10.1126/science.1092385 15001782

[147] Shaul ME Fridlender ZG. Cancer-related circulating and tumor-associated neutrophils – subtypes, sources and function. FEBS J. (2018) 285:4316–42. 10.1111/febs.14524 29851227

[148] Clark SR Ma AC Tavener SA McDonald B Goodarzi Z Kelly MM. Platelet TLR4 activates neutrophil extracellular traps to ensnare bacteria in septic blood. Nat Med. (2007) 13:463–9. 10.1038/nm1565 17384648

[149] El Rayes T Catena R Lee S Stawowczyk M Joshi N Fischbach C. Lung inflammation promotes metastasis through neutrophil protease-mediated degradation of Tsp-1. Proc Natl Acad Sci USA. (2015) 112:16000–5. 10.1073/pnas.1507294112 26668367 PMC4703007

[150] Park J Wysocki RW Amoozgar Z Maiorino L Fein MR Jorns J. Cancer cells induce metastasis-supporting neutrophil extracellular DNA traps. Sci Transl Med. (2016) 8:361ra138. 10.1126/scitranslmed.aag1711 27798263 PMC5550900

[151] Lee W Ko SY Mohamed MS Kenny HA Lengyel E Naora H. Neutrophils facilitate ovarian cancer premetastatic niche formation in the omentum. J Exp Med. (2019) 216:176–94. 10.1084/jem.20181170 30567719 PMC6314534

[152] Engblom C Pfirschke C Zilionis R Da Silva Martins J Bos SA Courties G Osteoblasts remotely supply lung tumors with cancer-promoting SiglecF(high) neutrophils. Science. (2017) 358:eaal5081 10.1126/science.aal5081 29191879 PMC6343476

[153] Colombo MP Lombardi L Stoppacciaro A Melani C Parenza M Bottazzi B. Granulocyte colony-stimulating factor (G-CSF) gene transduction in murine adenocarcinoma drives neutrophil-mediated tumor inhibition *in vivo*. Neutrophils discriminate between G-CSF-producing and G-CSF-nonproducing tumor cells. J Immunol. (1992) 149:113–9. 1376745

[154] Finisguerra V Di Conza G Di Matteo M Serneels J Costa S Thompson AR. MET is required for the recruitment of anti-tumoural neutrophils. Nature. (2015) 522:349–53. 10.1038/nature14407 25985180 PMC4594765

[155] Kousis PC Henderson BW Maier PG Gollnick SO. Photodynamic therapy enhancement of antitumor immunity is regulated by neutrophils. Cancer Res. (2007) 67:10501–10. 10.1158/0008-5472.CAN-07-1778 17974994 PMC2919236

[156] Fridlender ZG Sun J Kim S Kapoor V Cheng G Ling L. Polarization of tumor-associated neutrophil phenotype by TGF-beta: “N1” versus “N2” TAN. Cancer Cell. (2009) 16:183–94. 10.1016/j.ccr.2009.06.017 19732719 PMC2754404

[157] Andzinski L Kasnitz N Stahnke S Wu CF Gereke M von Kockritz-Blickwede M. Type I IFNs induce anti-tumor polarization of tumor associated neutrophils in mice and human. Int J Cancer. (2016) 138:1982–93. 10.1002/ijc.29945 26619320

[158] Zhang X Shi H Yuan X Jiang P Qian H Xu W. Tumor-derived exosomes induce N2 polarization of neutrophils to promote gastric cancer cell migration. Mol Cancer. (2018) 17:146. 10.1186/s12943-018-0898-6 30292233 PMC6174070

[159] Galon J Mlecnik B Bindea G Angell HK Berger A Lagorce C. Towards the introduction of the 'Immunoscore' in the classification of malignant tumours. J Pathol. (2014) 232:199–209. 10.1002/path.4287 24122236 PMC4255306

[160] Krieg C Nowicka M Guglietta S Schindler S Hartmann FJ Weber LM High-dimensional single-cell analysis predicts response to anti-PD-1 immunotherapy. Nat Med. (2018) 24:144–53. 10.1038/nm.4466 29309059

[161] Atanasov G Pötner C Aust G Schierle K Dietel C Benzing C. TIE2-expressing monocytes and M2-polarized macrophages impact survival and correlate with angiogenesis in adenocarcinoma of the pancreas. Oncotarget. (2018) 9:29715. 10.18632/oncotarget.25690 30038715 PMC6049857

[162] He YF Wang CQ Yu Y Qian J Song K Sun QM. Tie2-expressing monocytes are associated with identification and prognoses of hepatitis B virus related hepatocellular carcinoma after resection. PLoS ONE. (2015) 10:e0143657. 10.1371/journal.pone.0143657 26599011 PMC4658096

[163] Shoji H Yoshio S Mano Y Doi H Sugiyama M Osawa Y. Pro-angiogenic TIE-2-expressing monocytes/TEMs as a biomarker of the effect of sorafenib in patients with advanced hepatocellular carcinoma. Int J Cancer. (2017) 141:1011–7. 10.1002/ijc.30804 28555943

[164] Zhang QW Liu L Gong CY Shi HS Zeng YH Wang XZ. Prognostic significance of tumor-associated macrophages in solid tumor: a meta-analysis of the literature. PLoS ONE. (2012) 7:e50946. 10.1371/journal.pone.0050946 23284651 PMC3532403

[165] Guthrie GJ Roxburgh CS Farhan-Alanie OM Horgan PG McMillan DC. Comparison of the prognostic value of longitudinal measurements of systemic inflammation in patients undergoing curative resection of colorectal cancer. Br J Cancer. (2013) 109:24–8. 10.1038/bjc.2013.330 23799846 PMC3708558

[166] Wang J Jia Y Wang N Zhang X Tan B Zhang G. The clinical significance of tumor-infiltrating neutrophils and neutrophil-to-CD8+ lymphocyte ratio in patients with resectable esophageal squamous cell carcinoma. J Transl Med. (2014) 12:7. 10.1186/1479-5876-12-7 24397835 PMC3895663

[167] Nywening TM Belt BA Cullinan DR Panni RZ Han BJ Sanford DE. Targeting both tumour-associated CXCR2(+) neutrophils and CCR2(+) macrophages disrupts myeloid recruitment and improves chemotherapeutic responses in pancreatic ductal adenocarcinoma. Gut. (2018) 67:1112–23. 10.1136/gutjnl-2017-313738 29196437 PMC5969359

[168] Santoni M De Giorgi U Iacovelli R Conti A Burattini L Rossi L. Pre-treatment neutrophil-to-lymphocyte ratio may be associated with the outcome in patients treated with everolimus for metastatic renal cell carcinoma. Br J Cancer. (2013) 109:1755–9. 10.1038/bjc.2013.522 24008663 PMC3790174

[169] Leibowitz-Amit R Templeton A Omlin A Pezaro C Atenafu E Keizman D. Clinical variables associated with PSA response to abiraterone acetate in patients with metastatic castration-resistant prostate cancer. Ann Oncol. (2014) 25:657–62. 10.1093/annonc/mdt581 24458472 PMC4433513

[170] Lorente D Mateo J Templeton A Zafeiriou Z Bianchini D Ferraldeschi R. Baseline neutrophil–lymphocyte ratio (NLR) is associated with survival and response to treatment with second-line chemotherapy for advanced prostate cancer independent of baseline steroid use. Ann Oncol. (2014) 26:750–5. 10.1093/annonc/mdu587 25538172

[171] Jensen TO Schmidt H Moller HJ Hoyer M Maniecki MB Sjoegren P. Macrophage markers in serum and tumor have prognostic impact in American Joint Committee on Cancer stage I/II melanoma. J Clin Oncol. (2009) 27:3330–7. 10.1200/JCO.2008.19.9919 19528371

[172] Jensen TO Schmidt H Møller HJ Donskov F Høyer M Sjoegren P Christensen IJ. Intratumoral neutrophils and plasmacytoid dendritic cells indicate poor prognosis and are associated with pSTAT3 expression in AJCC stage I/II melanoma. Cancer. (2012) 118:2476–85. 10.1002/cncr.26511 21953023

[173] Trellakis S Farjah H Bruderek K Dumitru CA Hoffmann TK Lang S. Peripheral blood neutrophil granulocytes from patients with head and neck squamous cell carcinoma functionally differ from their counterparts in healthy donors. Int J Immunopathol Pharmacol. (2011) 24:683–93. 10.1177/039463201102400314 21978700

[174] Rao SP Sancho J Campos-Rivera J Boutin PM Severy PB Weeden T. Human peripheral blood mononuclear cells exhibit heterogeneous CD52 expression levels and show differential sensitivity to alemtuzumab mediated cytolysis. PLoS ONE. (2012) 7:e39416. 10.1371/journal.pone.0039416 22761788 PMC3382607

[175] Droeser RA Hirt C Eppenberger-Castori S Zlobec I Viehl CT Frey DM. High myeloperoxidase positive cell infiltration in colorectal cancer is an independent favorable prognostic factor. PLoS ONE. (2013) 8:e64814. 10.1371/journal.pone.0064814 23734221 PMC3667167

[176] Governa V Trella E Mele V Tornillo L Amicarella F Cremonesi E. The interplay between neutrophils and CD8(+) T cells improves survival in human colorectal cancer. Clin Cancer Res. (2017) 23:3847–58. 10.1158/1078-0432.CCR-16-2047 28108544

[177] Galdiero MR Bianchi P Grizzi F Di Caro G Basso G Ponzetta A. Occurrence and significance of tumor-associated neutrophils in patients with colorectal cancer. Int J Cancer. (2016) 139:446–56. 10.1002/ijc.30076 26939802

[178] Caruso RA Bellocco R Pagano M Bertoli G Rigoli L Inferrera C. Prognostic Value of intratumoral neutrophils in advanced gastric carcinoma in a high-risk area in Northern Italy. Modern Pathol. (2002) 15:831. 10.1097/01.MP.0000020391.98998.6B 12181268

[179] Carus A Ladekarl M Hager H Pilegaard H Nielsen PS Donskov F. Tumor-associated neutrophils and macrophages in non-small cell lung cancer: no immediate impact on patient outcome. Lung Cancer. (2013) 81:130–7. 10.1016/j.lungcan.2013.03.003 23540719

[180] Sunakawa Y Stintzing S Cao S Heinemann V Cremolini C Falcone A. Variations in genes regulating tumor-associated macrophages (TAMs) to predict outcomes of bevacizumab-based treatment in patients with metastatic colorectal cancer: results from TRIBE and FIRE3 trials. Ann Oncol. (2015) 26:2450–6. 10.1093/annonc/mdv474 26416897 PMC4658546

[181] Nywening TM Wang-Gillam A Sanford DE Belt BA Panni RZ Cusworth BM. Targeting tumour-associated macrophages with CCR2 inhibition in combination with FOLFIRINOX in patients with borderline resectable and locally advanced pancreatic cancer: a single-centre, open-label, dose-finding, non-randomised, phase 1b trial. Lancet Oncol. (2016) 17:651–62. 10.1016/S1470-2045(16)00078-4 27055731 PMC5407285

[182] Sandhu SK Papadopoulos K Fong PC Patnaik A Messiou C Olmos D. A first-in-human, first-in-class, phase I study of carlumab (CNTO 888), a human monoclonal antibody against CC-chemokine ligand 2 in patients with solid tumors. Cancer Chemother Pharmacol. (2013) 71:1041–50. 10.1007/s00280-013-2099-8 23385782

[183] Pienta KJ Machiels JP Schrijvers D Alekseev B Shkolnik M Crabb SJ. Phase 2 study of carlumab (CNTO 888), a human monoclonal antibody against CC-chemokine ligand 2 (CCL2), in metastatic castration-resistant prostate cancer. Invest New Drugs. (2013) 31:760–8. 10.1007/s10637-012-9869-8 22907596

[184] Brana I Calles A LoRusso PM Yee LK Puchalski TA Seetharam S. Carlumab, an anti-C-C chemokine ligand 2 monoclonal antibody, in combination with four chemotherapy regimens for the treatment of patients with solid tumors: an open-label, multicenter phase 1b study. Target Oncol. (2015) 10:111–23. 10.1007/s11523-014-0320-2 24928772

[185] Cannarile MA Weisser M Jacob W Jegg MA Ries CH Rüttinger D. Colony-stimulating factor 1 receptor (CSF1R) inhibitors in cancer therapy. J Immuno Ther Cancer. (2017) 5:53. 10.1186/s40425-017-0257-y 28716061 PMC5514481

[186] Cassier PA Italiano A Gomez-Roca CA Le Tourneau C Toulmonde M Cannarile MA. CSF1R inhibition with emactuzumab in locally advanced diffuse-type tenosynovial giant cell tumours of the soft tissue: a dose-escalation and dose-expansion phase 1 study. Lancet Oncol. (2015) 16:949–56. 10.1016/S1470-2045(15)00132-1 26179200

[187] DeNardo DG Brennan DJ Rexhepaj E Ruffell B Shiao SL Madden SF. Leukocyte complexity predicts breast cancer survival and functionally regulates response to chemotherapy. Cancer Discov. (2011) 1:54–67. 10.1158/2159-8274.CD-10-0028 22039576 PMC3203524

[188] Mok S Koya RC Tsui C Xu J Robert L Wu L. Inhibition of CSF-1 receptor improves the antitumor efficacy of adoptive cell transfer immunotherapy. Cancer Res. (2014) 74:153–61. 10.1158/0008-5472.CAN-13-1816 24247719 PMC3947337

[189] Zhu Y Knolhoff BL Meyer MA Nywening TM West BL Luo J. CSF1/CSF1R blockade reprograms tumor-infiltrating macrophages and improves response to T-cell checkpoint immunotherapy in pancreatic cancer models. Cancer Res. (2014) 74:5057–69. 10.1158/0008-5472.CAN-13-3723 25082815 PMC4182950

[190] Cuccarese MF Dubach JM Pfirschke C Engblom C Garris C Miller MA. Heterogeneity of macrophage infiltration and therapeutic response in lung carcinoma revealed by 3D organ imaging. Nat Commun. (2017) 8:14293. 10.1038/ncomms14293 28176769 PMC5309815

[191] Yan D Kowal J Akkari L Schuhmacher AJ Huse JT West BL. Inhibition of colony stimulating factor-1 receptor abrogates microenvironment-mediated therapeutic resistance in gliomas. Oncogene. (2017) 36:6049–58. 10.1038/onc.2017.261 28759044 PMC5666319

[192] Giustini N Bernthal NM Bukata SV Singh AS. Tenosynovial giant cell tumor: case report of a patient effectively treated with pexidartinib (PLX3397) and review of the literature. Clin Sarcoma Res. (2018) 8:14. 10.1186/s13569-018-0101-2 30002809 PMC6038319

[193] Ha H Debnath B Neamati N. Role of the CXCL8-CXCR1/2 axis in cancer and inflammatory diseases. Theranostics. (2017) 7:1543–88. 10.7150/thno.15625 28529637 PMC5436513

[194] Schott AF Goldstein LJ Cristofanilli M Ruffini PA McCanna S Reuben JM. Phase Ib pilot study to evaluate reparixin in combination with weekly paclitaxel in patients with HER-2-negative metastatic breast cancer. Clin Cancer Res. (2017) 23:5358–65. 10.1158/1078-0432.CCR-16-2748 28539464 PMC5600824

[195] Muller AJ DuHadaway JB Donover PS Sutanto-Ward E Prendergast GC. Inhibition of indoleamine 2,3-dioxygenase, an immunoregulatory target of the cancer suppression gene Bin1, potentiates cancer chemotherapy. Nat Med. (2005) 11:312. 10.1038/nm1196 15711557

[196] Iversen TZ Engell-Noerregaard L Ellebaek E Andersen R Larsen SK Bjoern J. Long-lasting disease stabilization in the absence of toxicity in metastatic lung cancer patients vaccinated with an epitope derived from indoleamine 2,3 dioxygenase. Clin Cancer Res. (2014) 20:221–32. 10.1158/1078-0432.CCR-13-1560 24218513

[197] Soliman HH Jackson E Neuger T Dees EC Harvey RD Han H. A first in man phase I trial of the oral immunomodulator, indoximod, combined with docetaxel in patients with metastatic solid tumors. Oncotarget. (2014) 5:8136–46. 10.18632/oncotarget.2357 25327557 PMC4226672

[198] Komiya T Huang CH. Updates in the clinical development of epacadostat and other indoleamine 2,3-dioxygenase 1 inhibitors (IDO1) for human cancers. Front Oncol. (2018) 8:423. 10.3389/fonc.2018.00423 30338242 PMC6180183

